# IL-1β and TNF-α play an important role in modulating the risk of periodontitis and Alzheimer’s disease

**DOI:** 10.1186/s12974-023-02747-4

**Published:** 2023-03-13

**Authors:** Rachel Pei-Hsuan Wang, Jianpan Huang, Kannie Wai Yan Chan, Wai Keung Leung, Tetsuya Goto, Yuen-Shan Ho, Raymond Chuen-Chung Chang

**Affiliations:** 1 Laboratory of Neurodegenerative Diseases, School of Biomedical Sciences, LKS Faculty of Medicine, The University of Hong Kong, Laboratory Block, Rm. L4-49, 21 Sassoon Road, Pokfulam, Hong Kong SAR, China; 2 Department of Biomedical Engineering, City University of Hong Kong, Hong Kong SAR, China; 3 Faculty of Dentistry, The University of Hong Kong, Hong Kong SAR, China; 4 Division of Oral Anatomy and Histology, Graduate School of Medical and Dental Sciences, Kagoshima University, Kagoshima, Japan; 5 School of Nursing, Faculty of Health and Social Sciences, Hong Kong Polytechnic University, Hunghom, Kowloon, Hong Kong SAR, China; 6 State Key Laboratory of Brain and Cognitive Sciences, The University of Hong Kong, Pokfulam, Hong Kong SAR, China

**Keywords:** Alzheimer’s disease, Aging, Cognitive dysfunctions, Neuroinflammation, Periodontitis

## Abstract

**Background:**

Systemic activation of the immune system can exert detrimental effects on the central nervous system. Periodontitis, a chronic disease of the oral cavity, is a common source of systemic inflammation. Neuroinflammation might be a result of this to accelerate progressive deterioration of neuronal functions during aging or exacerbate pre-existing neurodegenerative diseases, such as Alzheimer’s disease. With advancing age, the progressive increase in the body’s pro-inflammatory status favors the state of vulnerability to both periodontitis and Alzheimer’s disease. In the present study, we sought to delineate the roles of cytokines in the pathogenesis of both diseases.

**Methods:**

To examine the impacts of periodontitis on the onset and progression of Alzheimer’s disease, 6-month-old female 3 × Tg-AD mice and their age-matched non-transgenic mice were employed. Periodontitis was induced using two different experimental models: heat-killed bacterial-induced periodontitis and ligature-induced periodontitis. To delineate the roles of pro-inflammatory cytokines in the pathogenesis of periodontitis and Alzheimer’s disease, interleukin 1 beta (IL-1β) and tumor necrosis factor-alpha (TNF-α) were also injected into the buccal mandibular vestibule of mice.

**Results:**

Here, we show that IL-1β and TNF-α were two of the most important and earliest cytokines upregulated upon periodontal infection. The systemic upregulation of these two cytokines promoted a pro-inflammatory environment in the brain contributing to the development of Alzheimer’s disease-like pathology and cognitive dysfunctions. Periodontitis-induced systemic inflammation also enhanced brain inflammatory responses and subsequently exacerbated Alzheimer’s disease pathology and cognitive impairment in 3 × Tg-AD mice. The role of inflammation in connecting periodontitis to Alzheimer’s disease was further affirmed in the conventional magnetization transfer experiment in which increased glial responses resulting from periodontitis led to decreased magnetization transfer ratios in the brain of 3 × Tg-AD mice.

**Conclusions:**

Systemic inflammation resulting from periodontitis contributed to the development of Alzheimer’s disease tau pathology and subsequently led to cognitive decline in non-transgenic mice. It also potentiated Alzheimer’s disease pathological features and exacerbated impairment of cognitive function in 3 × Tg-AD mice. Taken together, this study provides convincing evidence that systemic inflammation serves as a connecting link between periodontitis and Alzheimer’s disease.

**Supplementary Information:**

The online version contains supplementary material available at 10.1186/s12974-023-02747-4.

## Background

Peripheral immune activation can have profound effects on the central nervous system (CNS), initiating or perpetuating neurodegenerative brain changes that underlie cognitive impairment and dementia. The impact of systemic inflammation on the brain has been affirmed in numerous and many of our studies. For instance, we have shown that subcutaneous injection of live *Escherichia coli* upregulated mRNA levels of interleukin 1 beta (IL-1β), tumor necrosis factor-alpha (TNF-α), interleukin 6 (IL-6), and cyclooxygenase-2 (COX-2) in the hippocampus and hypothalamus of mice, and induced sickness behavior within 4 h of infection [1]. We have also established an experimental model of laparotomy to examine the development of neuropathology and cognitive dysfunctions after surgery [2].

Here, we aimed to examine the impact of periodontitis on the onset and progression of Alzheimer’s disease (AD). Periodontitis is a pathological inflammatory condition of the teeth-supporting tissues characterized by the degradation of the connective tissue and alveolar bone resorption, ultimately resulting in tooth loss [3]. Many contributing inflammatory mediators have been associated with the pathogenesis and progression of periodontal diseases. Of note, IL-1β and TNF-α are key mediators of periodontal inflammation and bone destruction [4]. Studies have also shown that IL-1β and TNF-α salivary levels increased in diseased groups and decreased in response to periodontal therapy, providing support for their use as biomarkers for the detection of periodontal diseases [5].

Given that the inflammatory processes associated with periodontitis are not limited to the oral cavity, numerous studies have reported the association between periodontitis and neurodegenerative disorders, especially AD. In particular, a retrospective matched cohort study reported that a 10-year chronic periodontitis exposure was associated with an increased risk of developing AD [6]. Elevated serum antibodies to periodontal pathogens were also found to be present in AD patients at baseline, years before the diagnosis of neurological changes [7]. In addition, the presence of periodontitis was shown to associate with a sixfold increase in the rate of cognitive decline and a relative increase in the pro-inflammatory state in AD patients over a 6-month follow-up period [8]. Furthermore, another longitudinal study that followed 152 subjects for 20 years demonstrated that increased periodontal inflammation was correlated with lower cognitive levels [9]. These studies indicate that periodontitis contributes to the risk of AD onset and/or progression by modulating the host’s inflammatory state.

It has become increasingly apparent that AD pathophysiology entails dynamic inflammatory processes from both the peripheral and central immune compartments. In addition, the association between peripheral/central inflammatory mediators and cognitive functions has been documented in numerous studies. For instance, greater systemic levels of IL-6 and C-reactive protein (CRP) were found to associate with poorer cognitive performance and increased risk of dementia [10, 11]. Raised serum levels of IL-1β in AD patients without delirium were also associated with accelerated decline in cognition [12]. More recently, when assessing different cytokines in the blood of individuals along the AD continuum, IL-1β was found to be the variable most significantly associated with mild cognitive impairment-to-dementia conversion [13]. Similarly, TNF-α has also been implicated in memory deficits. In one longitudinal study, high baseline plasma TNF-α levels in combination with acute systemic inflammatory events were found to associate with a tenfold greater rate of cognitive decline over a half-year period [14]. Peripheral administration of a receptor targeting TNF-α also counteracted amyloid-induced memory impairment [15]. Based on the literature data available, we aimed to delineate the roles of IL-1β and TNF-α in the pathogenesis of periodontitis and AD.

Besides periodontitis, aging also leads to dysregulated immune-inflammatory responses [16]. Given that aging is a common risk factor shared by both periodontitis and AD, we hypothesized that cytokines circulating in the periodontium resulting from aging or bacterial infections could modulate cognitive functions and neuroimmune responses, contributing to the risk of AD.

## Materials and methods

### Mice

Female C57BL/6 J mice were purchased from the Centre for Comparative Medicine Research, The University of Hong Kong. Female homozygous 3 × Tg-AD mice (B6; 129-Psen1tm1Mpm Tg (APPSwe, tauP301L) 1L fa/MmJax) were bred from in-house colonies, established from original breeding pairs purchased from The Jackson Laboratory. Background strain- and age-matched female B6129SF2/J mice were used as non-transgenic (nTg) controls. To assess age-associated changes in the periodontium and the brain, female C57BL/6 J and 3 × Tg-AD mice at 3 months, 8 months, and 15–16 months of age were used. To examine the impacts of periodontitis and cytokines on the onset and progression of AD, 6-month-old female 3 × Tg-AD mice and their age-matched nTg mice were employed. Only female mice were used in this study as several lines of evidence suggest that the phenotype of female 3xTg-AD mice is consistent and shows a close association with that observed in AD patients and other animal models [17].

All animals were held in the Centre for Comparative Medicine Research (CCMR) of The University of Hong Kong, which is fully accredited by the Association for Assessment and Accreditation of Laboratory Animal Care International (AAALAC). Mice were housed in a temperature-controlled room at 20–22 °C, with humidity of 50 ± 10%, and were kept on a 12/12 h light/dark cycle. All mice had access to food and water ad libitum. Mice handling and all other procedures were conducted in accordance with the National Institutes of Health guide for the care and use of laboratory animals and the Animals (Control of Experiments) Ordinance, Hong Kong, China. The use of animals was approved by the Department of Health, Hong Kong, and the Committee on the Use of Live Animals in Teaching and Research, The University of Hong Kong (#4857-18 and #5238-19). All efforts were made to minimize animal numbers and suffering.

### Body weight measurements

Body weights for each mouse were measured weekly until the end of the experimental time point using a standard scale and were reported as percent change from baseline.

### Microcomputed tomography (micro-CT) scanning

After euthanasia, mandibular samples were dissected from mice and three-dimensional microcomputed tomography was performed using a micro-CT scanner (SkyScan 1172, Bruker, Kontich, Belgium). Scanning was performed using a source voltage of 80 kV with a beam current of 100 μA. A camera exposure time of 3630 ms, a rotation step of 1°, a frame average of 2, and median filtering of the data were applied, and X-rays were filtered with 0.5 mm aluminum. The acquired micro-CT images were then reconstructed using Skyscan NRecon software with the following parameters: 1% smoothing filter, 20% ring artifacts reduction, 30% beam-hardening correction, and an attenuation coefficient range of 0–0.19. After reconstruction, the 3D volume viewer program, DataViewer, was used to realign images and save the appropriate plane.

Periodontal bone loss was measured as reported [18]. Briefly, linear measurements from the cementoenamel junction (CEJ) to the alveolar bone crest (ABC) (CEJ–ABC) were taken in the interdental region between the first and second molars using the SkyScan CT-analyzer program, CTAn.

Assessment of bone mineral density (g cm^−3^) was based on a pair of calibration phantoms of calcium hydroxyapatite (CaHA) with concentrations of 0.25 and 0.75 g cm^−3^. This allowed for the quantitative measurement of bone density by interpolation between the two known densities. All bone mineral density analysis was performed on at least 50 reconstructed images.

### Total RNA extraction and reverse transcription

Total RNA was extracted from different brain regions of the left hemispheres (hippocampus, frontal cortex, and hypothalamus) and mandibular buccal gingiva using TRI Reagent®. In brief, mice were euthanized using carbon dioxide asphyxiation. Tissues from different brain regions and the gums were quickly removed, micro-dissected on ice, and snap-frozen in liquid nitrogen. Under RNase-free conditions, the tissues were mechanically homogenized using RNAiso plus (Takara, Japan) and resuspended in diethylpyrocarbonate (DEPC)-treated water. The quality of RNA samples was verified using Nanodrop One (Thermo Fisher Scientific, Waltham, MA) to ensure the A260/A280 ratios were within the range of 1.8 to 2.0. RNA samples (0.5 μg) were then subsequently used for reverse transcription using PrimeScript™ Master Mix Kit (Perfect Real Time) (Takara, Japan), and the cDNA samples were further diluted 10 times in DEPC-treated water.

### Real-time PCR

Five µl of diluted cDNA samples were amplified in triplicate using TB Premix Ex Taq™ II kit (Takara, Japan) and the CFX96 Touch two-color Real-Time PCR detection System (Bio-Rad, Hercules, USA). The amplification conditions were as follows: activation at 95 °C for 30 s, followed by 41 cycles of denaturation at 95 °C for 5 s, and extension at 60 °C for 30 s. A melting curve analysis was subsequently performed which began at 55 °C for 5 s and increased to 95 °C in 0.5 °C increments. The relative expression levels of each cytokine were then normalized to those of the housekeeping gene glyceraldehyde 3-phosphate dehydrogenase (GAPDH) following the 2-ΔΔCt method [19].

To determine the total bacterial load, 100 ng of each DNA sample was used to amplify the 16S ribosomal RNA (16S rRNA) gene. The amplification conditions were the same as described above, except that the extension temperature was 58 °C. Quantification of the total bacterial load from the DNA samples was calculated using the standard curve generated from serial dilutions of known concentration of genomic DNA isolated from a pure culture of *Pg* and the results were reported as log(copies/μl).

All primer sequences used in this study are listed in Additional file 1: Table S1.

### Bacterial-induced periodontitis

Chronic periodontitis was induced by *Porphyromonas gingivalis* (*Pg*) (ATCC 33277) obtained from co-investigator Professor Wai Keung Leung at the Faculty of Dentistry, The University of Hong Kong. *Pg* was grown at 37 °C for 5 days in Trypticase soy broth (Becton Dickinson, Sparks, MD, USA) supplemented with yeast extract (5 mg/ml; Becton Dickinson, Sparks, MD, USA), hemin (5 mg/ml; Sigma Aldrich, St. Louis, MO, USA), and vitamin K (0.5 mg/ml; Sigma Aldrich, St. Louis, MO, USA). The bacteria were next harvested by centrifugation (5000 rpm), washed with phosphate-buffered saline (PBS) twice, and re-suspended in PBS. All reagents used were pre-reduced by incubating them in the anaerobic chamber overnight before use. The titer of *Pg* bacteria was determined with a spectrophotometer while an optical density of 660 nm equals 2 indicates *Pg* at 10^11^–10^12^ CFU/ml. To prepare for heat-killed bacteria, the bacterial cells re-suspended in PBS were heated up at 80 °C for 30 min. To induce chronic periodontitis, 6-month-old female 3 × Tg-AD mice and their age-matched nTg mice were randomly assigned to control and *Pg*-injected groups with 16 mice per group. Mice were bilaterally injected at the buccal of mandibular molars with 2 μl of heat-killed *Pg* using a 2.5 μl Hamilton syringe three times per week every other week for a total of 5 weeks. The control mice were injected with 2 μl of PBS. All injections were performed under anesthesia using intraperitoneal injection of ketamine/xylazine mixture (100 mg/kg ketamine/10 mg/kg xylazine). At the end of the experimental time point, i.e., by age of 7.5 months, the mandibles from each mouse were dissected and subjected to alveolar bone measurements via microcomputed tomography (Micro-CT) scanning.

### Cytokine injections

Recombinant murine IL-1β (211-11B, PeproTech, Rocky Hill, NJ, USA) and TNF-α (315-01A, PeproTech, Rocky Hill, NJ, USA) were reconstituted in sterile MilliQ water, according to the manufacturer’s instructions. The endotoxin levels for both recombinant cytokines were guaranteed to have an endotoxin level of less than 1 EU/μg, which were measured using the Charles River Endosafe–PTS Chromogenic LAL system. To induce periodontal inflammation, 6-month-old mice were randomly assigned to control and cytokine-injected groups with five mice per group. Bilateral injections (2.5 μl each) of mixed cytokines consisting of IL-1β (8.3 ng; 4150 U) and TNF-α (16.7 ng; 167 U) were performed in the mandibular buccal vestibule of mice using a 2.5 μl Hamilton syringe three times per week every other week for a total of 5 weeks. Control mice were injected with sterile MilliQ water. All injections were performed under anesthesia using intraperitoneal injection of ketamine/xylazine mixture (100 mg/kg ketamine/10 mg/kg xylazine). The amounts of cytokines selected were based on our own preliminary data demonstrating similar fold-change in cytokine expression in the gum tissues of mice injected with heat-killed *Pg*.

### Ligature-induced periodontitis

To induce chronic periodontitis, 6-month-old female 3 × Tg-AD mice and their age-matched nTg mice were randomly assigned to control and ligature groups with 19 mice per group. 5–0 silk ligatures were placed around the maxillary second molars of 6-month-old mice under anesthesia using intraperitoneal injection of ketamine/xylazine mixture (100 mg/kg ketamine/10 mg/kg xylazine). The ligatures were inspected once weekly and maintained in place for a total of 5 weeks throughout the experimental period. The control mice were anesthetized without undergoing ligature placement. At the end of the experimental timepoint, i.e., by age of 7.5 months; the maxilla from each mouse was dissected for macroscopic bone loss analysis, as previously described [20].

### Macroscopic bone loss analysis

After euthanasia, the maxilla from each mouse was dissected and immersed in 30% hydrogen peroxide overnight to completely remove soft tissue. The jaws were then dried at room temperature and stained with 0.1% methylene blue (Sigma Aldrich, St. Louis, USA) for 3–5 min. Excess methylene blue was removed using distilled water. Digital photographs of both buccal and palatal surfaces of the stained jaws were taken with Zeiss Axiocam 208 color using a 0.5x/0.125 Zeiss Plan-Apochromat objective. The distance between CEJ and ABC was measured at nine sites for each of the palatal and buccal surfaces of the maxillae using Image J Software (National Institutes of Health, USA).

### DNA extraction

Genomic DNA was extracted from freshly harvested gum and whole brain tissues, recovered ligatures, and *P. gingivalis* bacterial suspension cultures under aseptic conditions using the QiaAmp DNA Mini Kit (Qiagen Hong Kong Pte. Ltd, Hong Kong) according to the manufacturer’s instructions and quantified with Nanodrop One (Thermo Fisher Scientific, Waltham, MA). Samples were stored at − 20 °C until analysis.

### C-reactive protein ELISA

Protein levels of C-reactive protein (CRP) in the plasma were measured using a commercialized mouse CRP enzyme-linked immunosorbent assay (ELISA) kit (LS-F4264, Lifespan Bio, Seattle, WA, USA) according to the manufacturer’s instructions. Briefly, 0.8–1.0 ml of blood samples from the mouse were collected by cardiac puncture and collected in tubes containing 10 μl of 0.5 M EDTA. The tubes were centrifuged for 15 min at 1400 × *g* at 4 °C to obtain plasma. The obtained plasma was then stored at − 80 °C until analysis.

### Immunofluorescence staining

Following euthanasia by CO_2_ asphyxiation, mice were transcardially perfused with ice-cold saline. The brains were carefully removed and the right hemispheres were post-fixed for 72 h in 4% paraformaldehyde at 4 °C, and then dehydrated with a graded series of ethanol, cleared in xylene, and embedded in paraffin. 6 um-thick coronal brain sections were made for immunofluorescent staining. In brief, brain sections were dewaxed in two changes of xylene, followed by two changes of 100%, 96%, and 70% ethanol each. Sections were then rinsed thrice in tap water followed by immersion in distilled water. Antigen retrieval was achieved by incubating the sections in 10 mM citrate buffer with 0.1% Tween-20 (pH 6.0) for 15 min at 90˚C followed by cooling to room temperature. After washing with PBS, sections were blocked with 10% normal goat serum (Vector Laboratories, Inc., Burlingame, CA) for 1 h at room temperature and then incubated with primary antibody diluted in PBS containing 0.3% Triton X-100 at 4 °C overnight. For β-amyloid (Aβ) staining, sections were pre-treated with 90% formic acid for 5 min before blocking with 10% normal goat serum.

On the next day, sections were incubated with the corresponding Alexa Fluor® (Alexa-fluor 488, Alexa-fluor 568; Thermo Fisher Scientific, Waltham, MA) conjugated secondary antibody in DAKO antibody diluent (DAKO, Glostrup, Denmark) for 1 h at room temperature, counterstained with 2 μM 4',6-diamidino-2-phenylindole (DAPI) for 10 min at room temperature and mounted with coverslips using fluorescence mounting medium (DAKO, Glostrup, Denmark).

The primary antibodies used in the present study were as follows: rabbit anti-phosphorylated (p)-Tau (Ser396) (1:200, 44-752G, Thermo Fisher Scientific, Waltham, MA, USA), rabbit anti-phosphorylated (p)-Tau (Ser404) (1:100, 44-758G, Thermo Fisher Scientific, Waltham, MA, USA), mouse anti-glial fibrillary acidic protein (GFAP) (1:400, G3893, Sigma Aldrich, St. Louis, MO, USA), rabbit anti-tyrosine hydroxylase (TH) (1:1000, AB152, Sigma Aldrich, St. Louis, MO, USA), and mouse anti-β-amyloid (6E10) (1:400, 803004, Biolegend, San Diego, CA, USA). For the labeling of microglia, anti-ionized calcium-binding adaptor molecule 1 (Iba-1) antibodies of two providers and different host species were used: rabbit anti-Iba-1 (1:400, 019-19741, Wako, Chuo-ku, OSA, Japan) and mouse anti-Iba-1/AIF-1 (1:400, MABN92, Sigma Aldrich, St. Louis, MO, USA).

Confocal imaging was performed using a laser scanning confocal fluorescence microscope (Carl Zeiss LSM 800, Germany) equipped with ZEN light software at 1024 × 1024 resolution. The objective lenses used were Plan-Apochromat 10x (NA 0.45) Ph1 M27 and Plan-Apochromat 20x (NA 0.8) Ph2 M27. Z-stack images were acquired for each region and quantification of the images was performed using ImageJ software (National Institute of Health, USA) [21].

### Open field test

To test for sickness behavior and exploratory motivation, the mice were subjected to the open field test. The test was performed as previously described [22]. For this test, each mouse was placed in the center of an empty 40 × 40 cm square arena and allowed to freely explore for 10 min. During this period, the animal behavior was recorded by a video camera mounted overhead. Video tracking software (SMART 3.0, Panlab SL, Barcelona, Spain) was used for data analysis. During the analysis, the arena was divided into 25 zones (16 peripheral and 9 central). The total distance traveled, and the duration of time spent in the center and outer zones were measured.

### Spontaneous Y-maze test

The spontaneous Y-maze test was used to assess spatial working memory. For this test, each mouse was placed in one arm and allowed to freely explore for 5 min. During this period, the animal behavior was recorded by a video camera mounted overhead. The number of arm entries and triads was recorded to calculate the % of spontaneous alternation. An entry occurred when all four limbs were within one arm.

### Puzzle box test

To assess executive functions and cognitive ability, the puzzle box test was performed. The arena consisted of two compartments separated by a removable barrier: a brightly lit start zone and a smaller covered goal zone. The protocol was slightly modified from Abdallah et al. [23]. Briefly, each mouse was initially introduced into the start zone and trained to move into the goal zone through a narrow underpass. Mice were subjected to a total of seven trials (T1–T7) over three consecutive days with three trials on the first two days and one trial on the last day. During this period, the entrance to the goal zone was blocked with different objects with increasing difficulties to solve. The time required for each mouse to enter the goal zone was recorded.

### MRI experiments

MRI experiments were conducted at the City University of Hong Kong using a horizontal bore 3 Tesla Bruker BioSpec animal scanner (Bruker, Ettlingen, Germany). An 82 mm quadrature volume coil was used as a transmitter and a single surface coil was used as a receiver. Mice were first anesthetized using 2% isoflurane in concentrated oxygen before MRI and then maintained using 1–1.5% isoflurane during the MRI. After anesthesia, mice were placed on an animal bed with their head positioned using a bite bar. The body temperature was maintained at 37 °C using a warming pad connected to a water-heating system and the respiration was monitored by a respiratory sensor connected to a monitoring system. The image slice selection was achieved by collecting a central sagittal image of the mouse brain and setting the position of the coronal image slice to − 1.5 mm with respect to the anterior commissure (AC). Before magnetization transfer (MT) imaging, the B_0_ field over the mouse brain was shimmed using field-mapping and second-order shimming. The MT imaging sequence was rapid acquisition with refocused echoes (RARE) with a continuous-wave pre-saturation pulse. Images without pre-saturation (*M*_0_) and with pre-saturation at 0.6 kHz (*M*_sat_) were acquired for MT ratio (MTR) calculation: MTR = 1 − *M*_sat_/*M*_0_. The power and duration of the pre-saturation pulse were 0.6 µT and 3 s, respectively. Other imaging parameters were set as followings: repetition time (TR) = 5 s, echo time (TE) = 6 ms, RARE factor = 32, centric encoding, field of view (FOV) = 18 × 18 mm^2^, matrix size = 96 × 96, slice thickness = 2 mm.

### Statistical analysis

Statistical analysis was performed with Prism 8.0 (GraphPad Software, USA) using either unpaired *t* test, one-way or two-way analysis of variance (ANOVA) with Bonferroni’s or Tukey’s post-hoc test, wherever applicable. All results are expressed as mean ± SEM. *p* < 0.05 was considered statistically significant for all analyses.

## Results

### Aged mice exhibited significantly reduced % remaining periodontal bone and increased cytokine immune responses in the gums and the brains

**Fig. 1:**
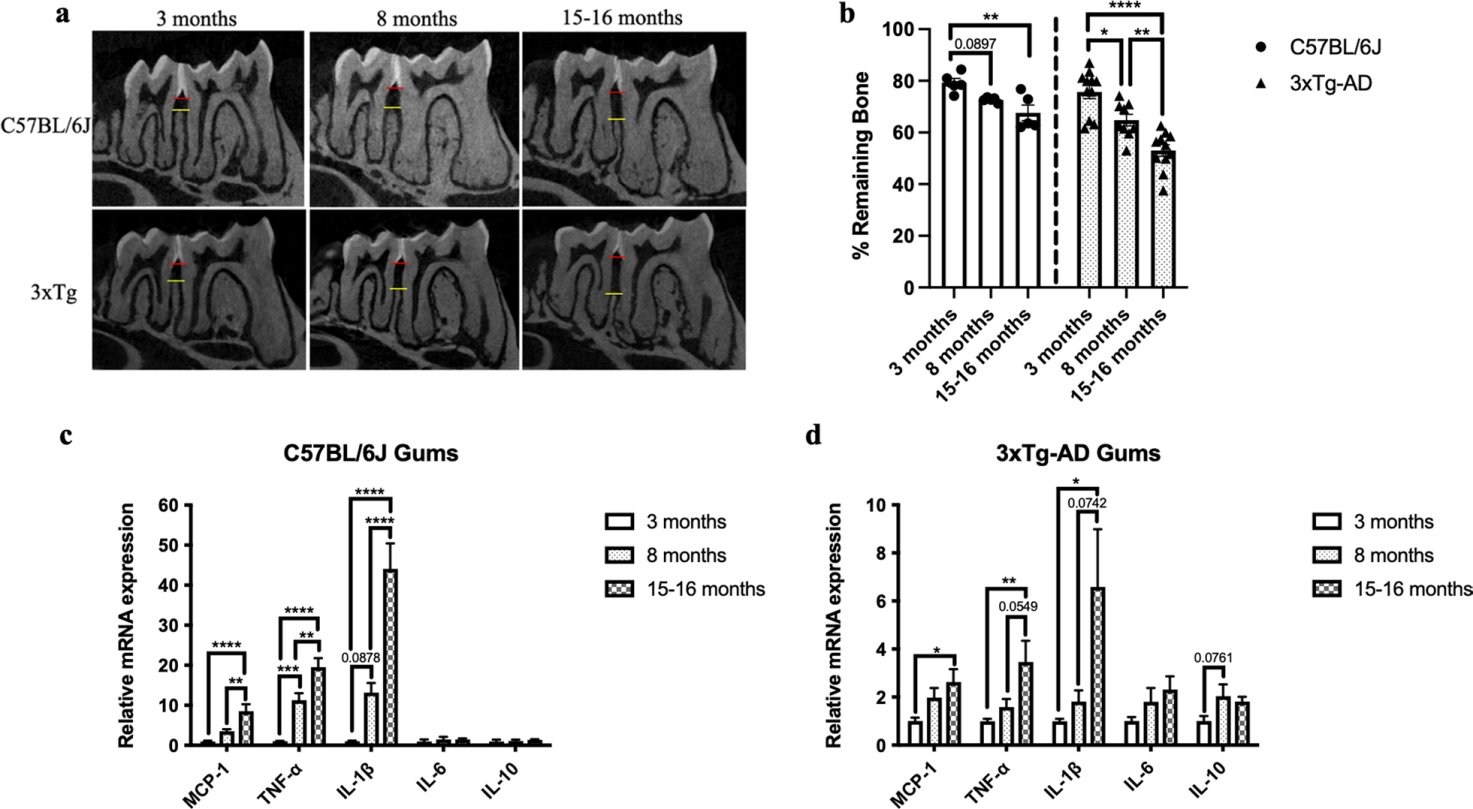

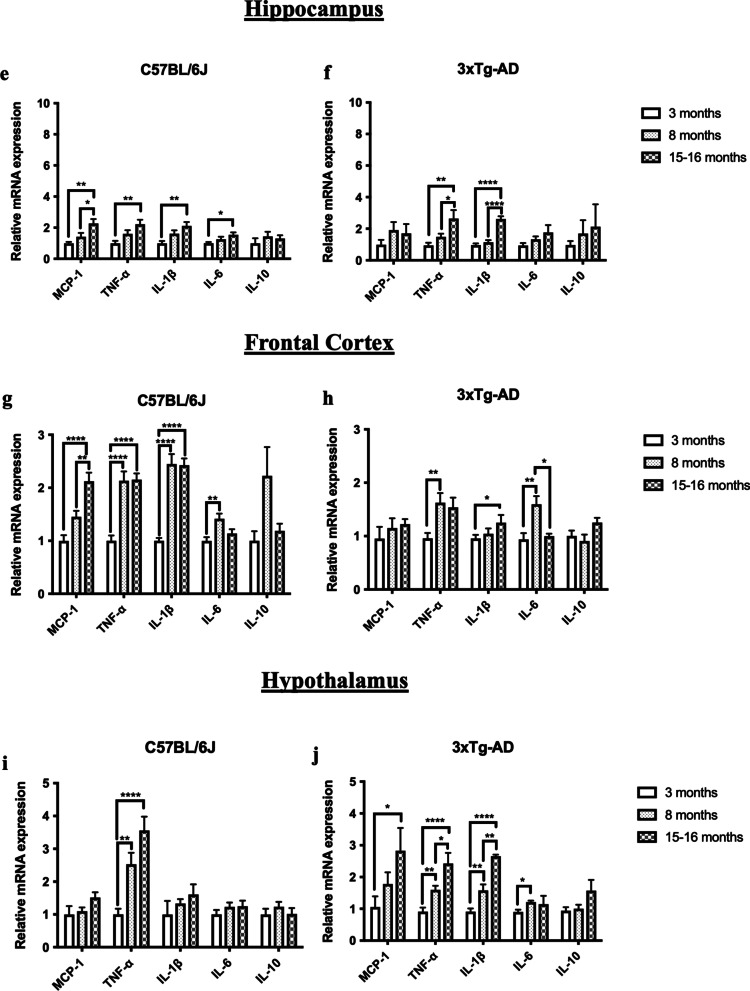
Age-dependent increased periodontal bone loss and increased cytokine immune responses in gums and brain specimens. **a** Representative two-dimensional sagittal micro-CT images of the left mandibular jaws from C57BL/6 J and 3 × Tg-AD mice at the ages investigated. Red and yellow bars indicate the distance from the cementoenamel junction (CEJ) to the alveolar bone crest (ABC). **b** Quantitative measurements of the vertical bone loss (mm) from CEJ to ABC between the first and second molars. Data are presented as mean ± SEM (C57BL/6 J mice: *n* = 5/age group, 3 × Tg-AD mice: *n* = 9–11/age group). Statistical analysis was done using two-way ANOVA with Tukey’s post-hoc test. **c**, **d** Relative mRNA expression levels of inflammatory mediators in the gums of (**c**) C57BL/6 J and (**d**) 3 × Tg-AD mice at different ages. Data are presented as mean ± SEM (C57BL/6 J mice: *n* = 10/age group, 3 × Tg-AD mice: *n* = 9–10/age group). **e**, **f** Relative mRNA expression levels of inflammatory mediators in the hippocampus of (**e**) C57BL/6 J and (**f**) 3 × Tg-AD mice at different ages. **g**, **h** Relative mRNA expression levels of inflammatory mediators in the frontal cortex of (**g**) C57BL/6 J and (**h**) 3 × Tg-AD mice at different ages. **i**, **j** Relative mRNA expression levels of inflammatory mediators in the frontal cortex of (**i**) C57BL/6 J and (**j**) 3 × Tg-AD mice at different ages. Data are presented as mean ± SEM (C57BL/6 J mice: *n* = 10/age group, 3 × Tg-AD mice: *n* = 4–10/age group). Statistical analysis was done using one-way ANOVA with Tukey’s post-hoc test. *p < 0.05, **p < 0.01, ***p < 0.001, ****p < 0.0001

Aging is associated with the occurrence and severity of periodontal destruction. By employing micro-computed tomography (Micro-CT) scan, our data revealed an age-associated increase in alveolar bone loss in C57BL/6 J and 3 × Tg-AD mice (Fig. 1a, b). Osteoporosis is a skeletal disorder clinically marked by the reduction of bone mineral density and degradation of bone tissue, leading to fragile bones with an increased risk of bone breakage. Both periodontal diseases and osteoporosis share characteristic features of bone loss. To determine whether the age-dependent loss of bone mineral density could contribute to the observed reduction of periodontal bone level in aged mice, bone mineral densities of the mandibular jaws were also evaluated. The observed periodontal bone loss occurred without the deterioration of the mouse mandibular second molar furcal alveolar bone mineral density (Additional file 1: Fig. S1). Consistently, the age-associated periodontal bone loss was accompanied by increased gene expression levels of MCP-1, IL-1β, and TNF-α in the gums (Fig. 1c, d). As neuroinflammation is well-known to be one of the neurobiological changes that occur in aging, we also examined the gene expression levels of inflammatory cytokines in the hippocampus, frontal cortex, and hypothalamus of mice at the ages followed. As expected, both mouse strains exhibited an age-related inflammatory phenotype in the brain (Fig. 1e–j).

### Systemic inflammation induced by periodontitis resulted in increased neuroimmune responses and modulated cognition in non-transgenic mice

**Fig. 2:**
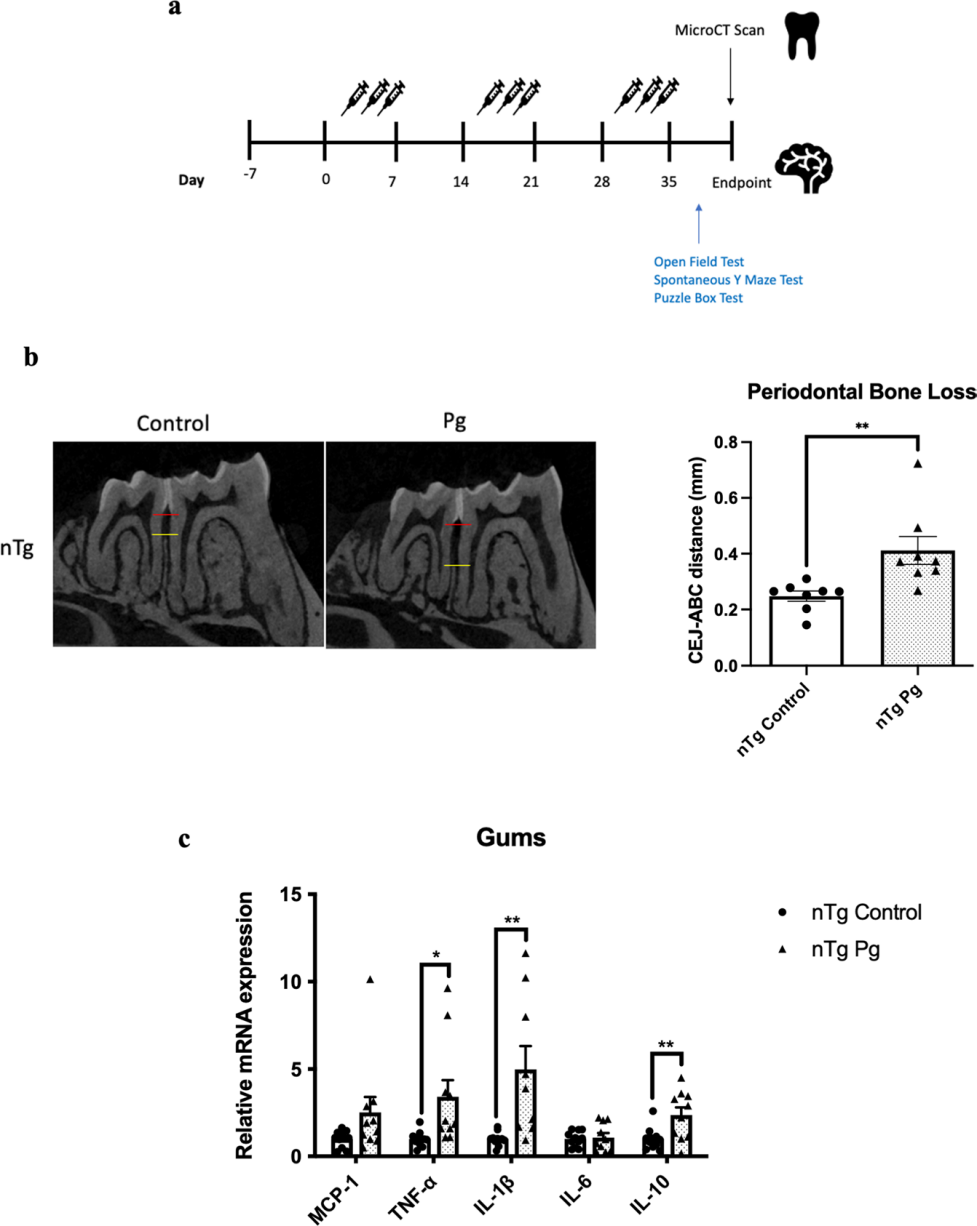

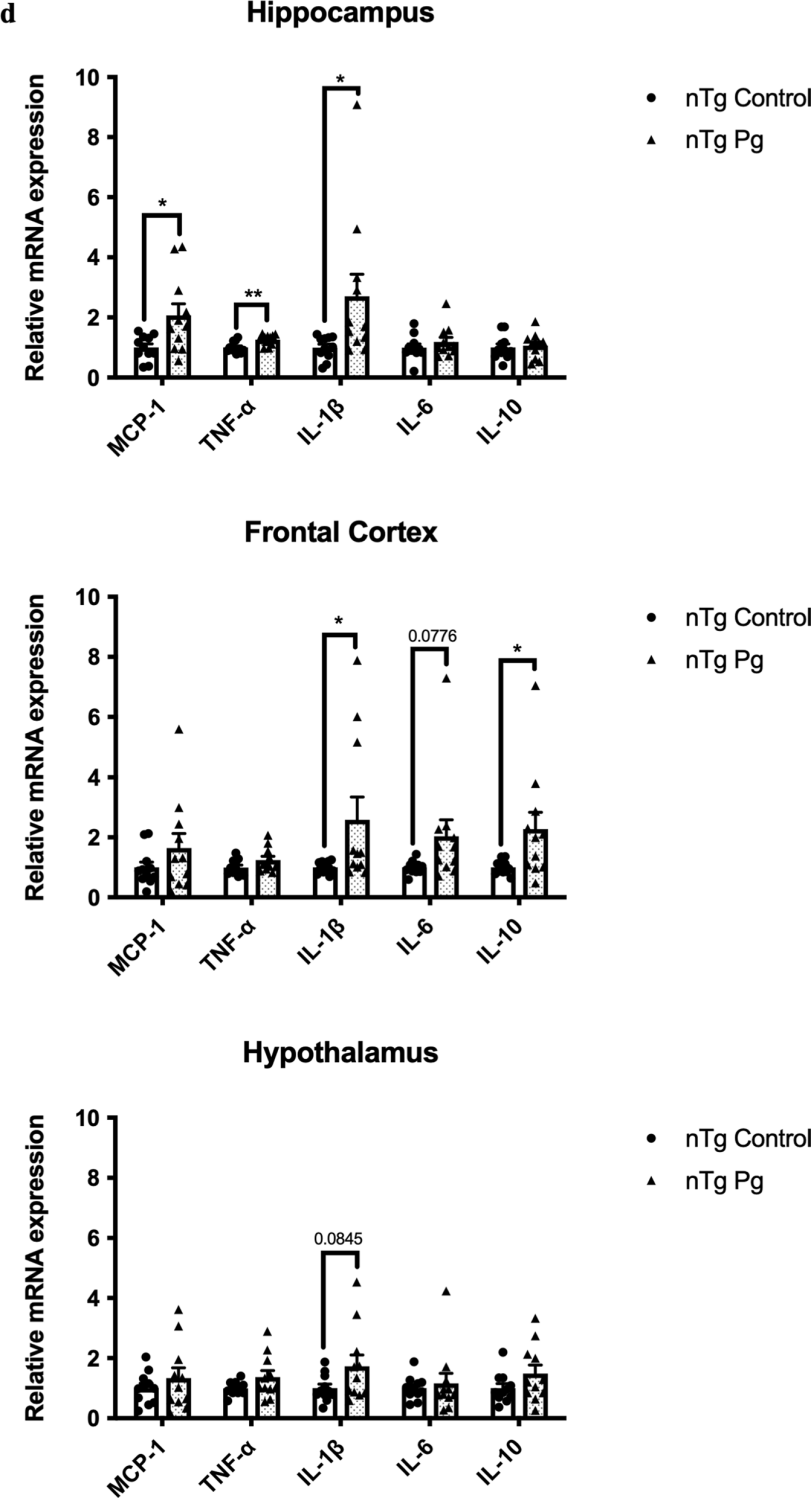

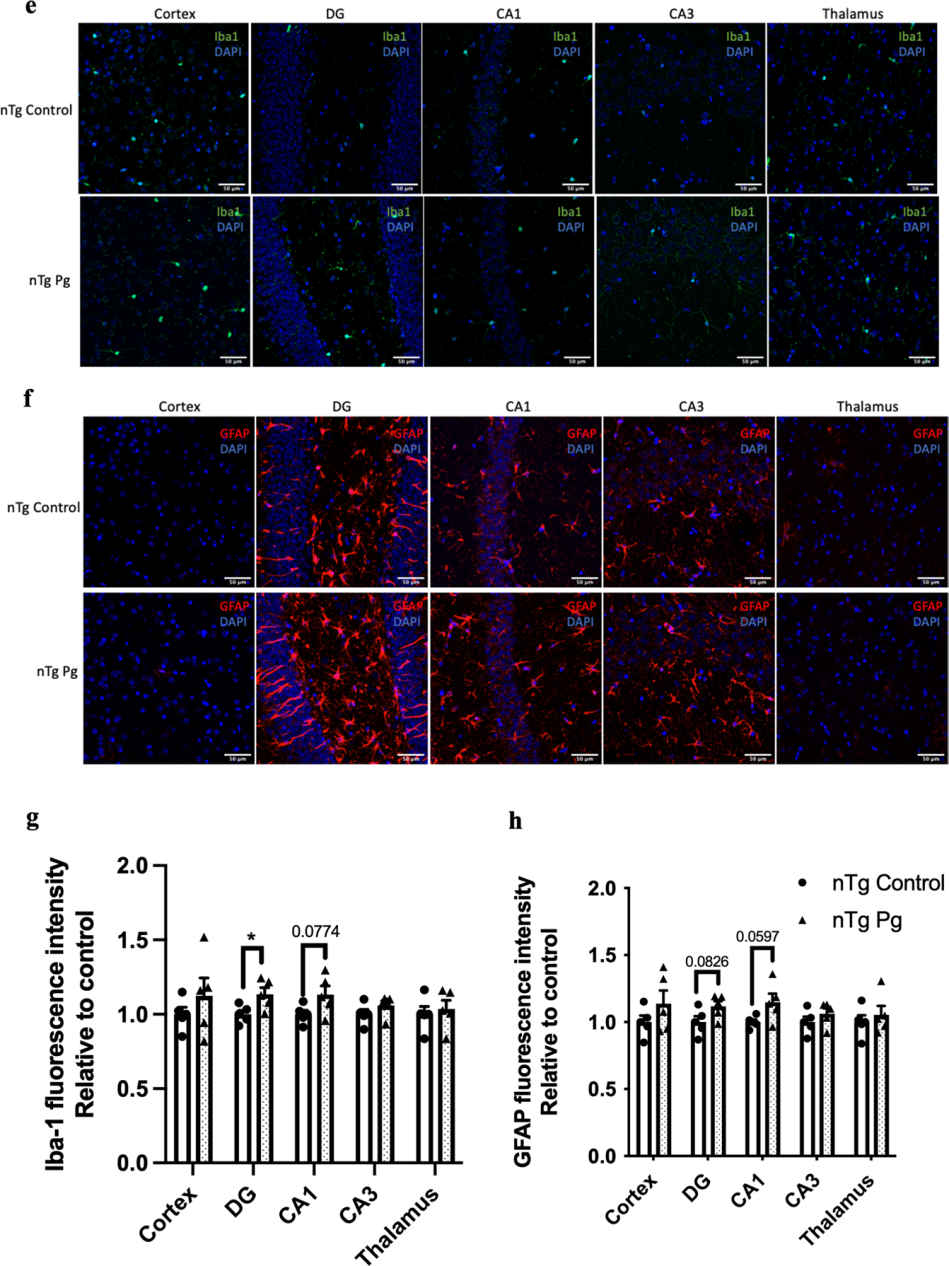
Bacterial-induced periodontitis increased immune responses in the gums and the brains of nTg mice. **a** Schematic illustration of the experimental timeline and treatment. **b** Representative two-dimensional sagittal images of the left mandibular jaws from nTg mice in each group. Red and yellow bars indicate the distance from the cementoenamel junction (CEJ) to the alveolar bone crest (ABC) (Left); Quantitative measurements of the vertical bone loss (mm) from CEJ to ABC between the first and second molars (Right). Data are presented as mean ± SEM (*n* = 8). **c** Relative mRNA expression levels of inflammatory mediators in the gums of nTg mice in each group. Data are presented as mean ± SEM (*n* = 10–11). **d** Relative mRNA expression levels of inflammatory mediators in the hippocampus, frontal cortex, and hypothalamus of nTg mice in each group. Data are presented as mean ± SEM (*n* = 11). **e** Representative images of immunofluorescence staining for Iba-1-positive microglia (green) and DAPI (blue) in the cortex, sub-regions of the hippocampus, and thalamus of nTg mice in each group. **f** Representative images of immunofluorescence staining for GFAP-positive astrocytes (red) and DAPI (blue) in the cortex, sub-regions of the hippocampus, and thalamus of nTg mice in each group. **g**, **h** Quantification of (**g**) Iba-1 and (**h**) GFAP immunofluorescence intensity in the cortex, sub-regions of the hippocampus, and thalamus of nTg mice in each group. Data are presented as mean ± SEM (*n* = 5). Statistical analysis was done using unpaired *t* test. *p < 0.05, **p < 0.01

Given that aging led to elevated levels of cytokines in the gums and the brains of mice, we next asked whether periodontitis-induced periodontal inflammation would modulate the risk of AD. Non-transgenic (nTg) mice were injected with heat-killed *P. gingivalis* (*Pg*) into the mandibular buccal vestibule (Fig. 2a). Injection of heat-killed *Pg* resulted in periodontal bone loss and systemic inflammation as indicated by elevated gene expression levels of IL-1β, TNF-α, and IL-10 in the gums (Fig. 2b, c) and CRP levels in the plasma (Additional file 1: Fig. S2a). Elevated levels of inflammatory cytokines in different brain regions were also observed (Fig. 2d). It has been well-documented that activation of glial cells is the primary source of cytokine production in the brain. To investigate this, double-immunolabeling of brain sections using ionized calcium-binding adapter molecule 1 (Iba-1) (WAKO) and glial fibrillary acidic protein (GFAP) was performed. By assessing Iba-1 and GFAP immunoreactivities in the brain, the fluorescence intensity of Iba-1 was found to be significantly increased in the dentate gyrus region and marginally increased in the CA1 region of *Pg*-injected mice (Fig. 2e, g). The fluorescence intensity of GFAP was also marginally increased in the dentate gyrus and CA1 regions of *Pg*-injected mice (Fig. 2f, h). We next evaluated the effects of periodontal inflammation on tau phosphorylation. Significantly increased immunoreactivities of tau396 and tau404 were observed in the brains of *Pg*-injected mice compared to control (Additional file 1: Fig. S3a–d).

The behavior of mice was next assessed. The % weight changes from baseline during the experimental period are shown in Additional file 1: Fig. S4. For the assessment of general exploratory and anxiety-like behavior in mice, the open field test was performed. No significant difference in the total distance traveled was found between the uninfected and infected mice, suggesting that *Pg*-induced periodontitis did not alter exploratory behavior in mice (Additional file 1: Fig. S5a). In addition, there was no difference in the % of time spent in the center between the groups, suggesting that both groups did not exhibit anxiety-like behavior (Additional file 1: Fig. S5b). Next, mice were subjected to spontaneous Y maze and puzzle box tests to assess their cognitive functions. Spontaneous alternation in the Y maze, an index of short-term spatial working memory in mice, was not significantly altered in both groups (Additional file 1: Fig. S5c, d). The puzzle box test is an assay for testing problem-solving ability and executive functions. No significant alterations in the problem-solving ability and short-term memory were observed in both groups. However, compared to uninfected mice, infected mice exhibited a significantly longer latency to reach the goal zone 24 h after exposure to the burrowing task (Additional file 1: Fig. S5e), suggesting that *Pg*-induced periodontitis led to impairment of long-term memory in nTg mice.

### IL-1β and TNF-α play a pivotal role in modulating the risk of periodontitis and AD

**Fig. 3:**
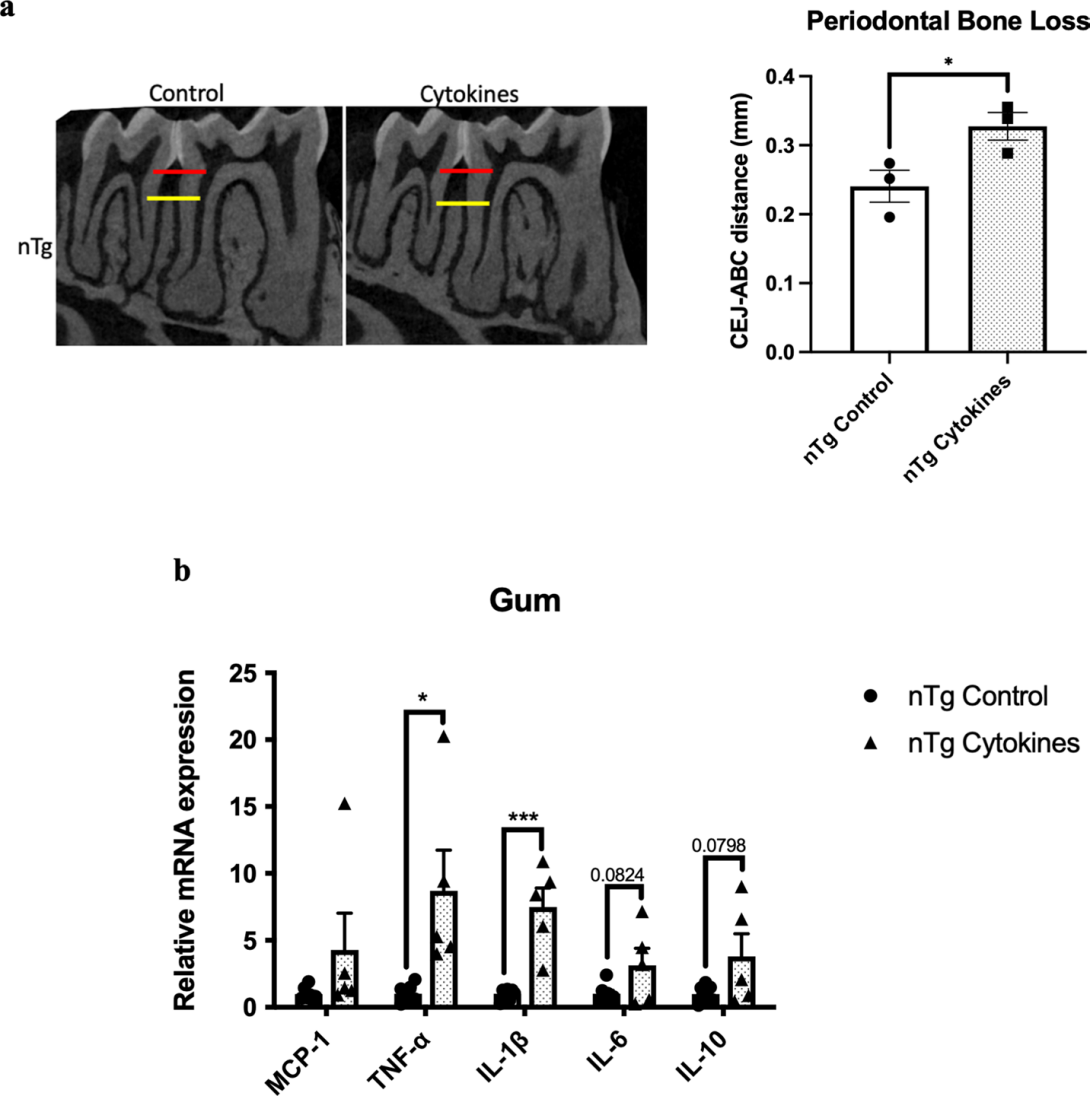

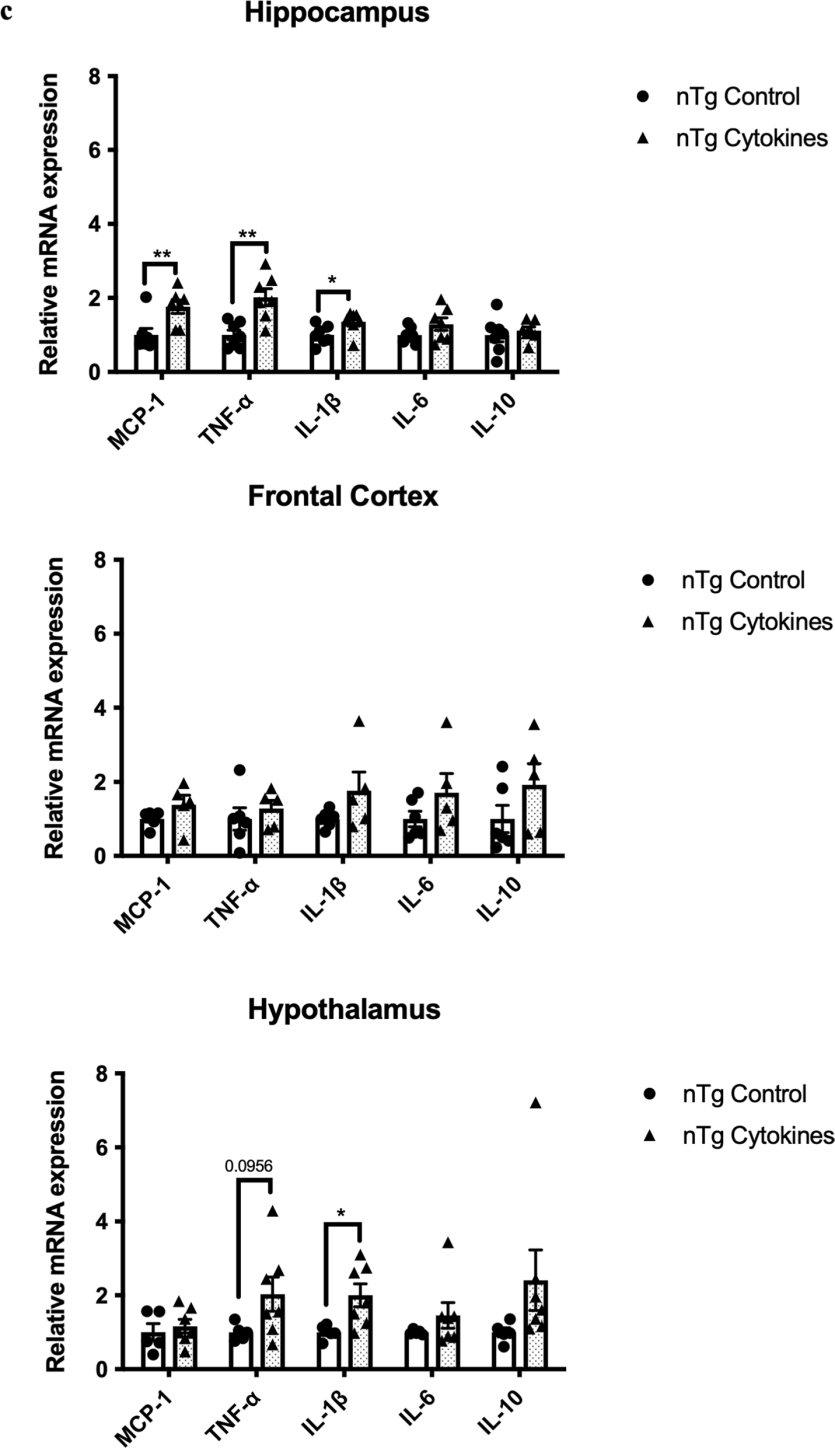

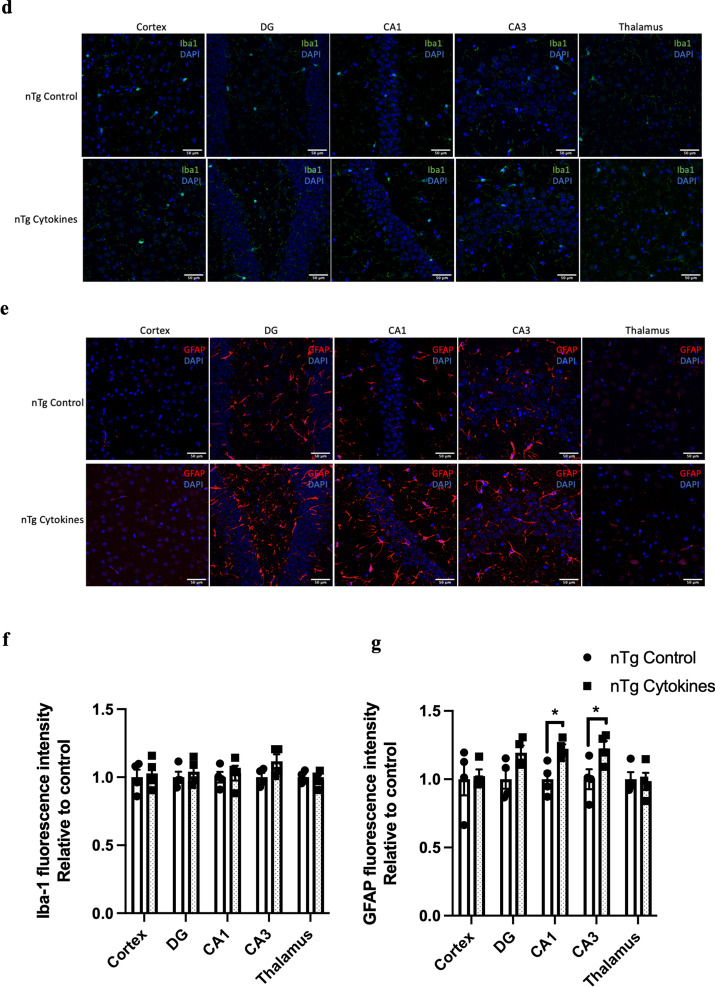
Mixed cytokines injections induced periodontal bone loss and increased immune responses in nTg mice. **a** Representative two-dimensional sagittal images of the left mandibular jaws from nTg mice in each group. Red and yellow bars indicate the distance from the cementoenamel junction (CEJ) to the alveolar bone crest (ABC) (Left); Quantitative measurements of the vertical bone loss (mm) from CEJ to ABC between the first and second molars (Right). Data are presented as mean ± SEM (*n* = 4). **b** Relative mRNA expression levels of inflammatory mediators in the gums of nTg mice in each group. **c** Relative mRNA expression levels of inflammatory mediators in the hippocampus, frontal cortex, and hypothalamus of nTg mice in each group. Data are presented as mean ± SEM (*n* = 5–7). **d** Representative images of immunofluorescence staining for Iba-1-positive microglia (green) and DAPI (blue) in the cortex, sub-regions of the hippocampus, and thalamus of nTg mice in each group. **e** Representative images of immunofluorescence staining for GFAP-positive astrocytes (red) and DAPI (blue) in the cortex, sub-regions of the hippocampus, and thalamus of nTg mice in each group. **f**, **g** Quantification of (**f**) Iba-1 and (**g**) GFAP immunofluorescence intensity in the cortex, sub-regions of the hippocampus, and thalamus of nTg mice in each group. Data are presented as mean ± SEM (*n* = 4). Statistical analysis was done using unpaired *t* test. *p < 0.05, **p < 0.01, ***p < 0.001

The upregulation of IL-1β and TNF-α levels in the gums of aged mice and *Pg*-injected mice led us to next delineate the roles of these two cytokines in the pathogenesis of periodontitis and AD. Injections of mixed cytokines in the mandibular buccal vestibule of nTg mice-induced periodontal bone loss, which was accompanied by elevated gene expression levels of IL-1β and TNF-α in the gums (Fig. 3a, b). It also led to elevated gene expression levels of MCP-1, IL-1β, and TNF-α in the hippocampus and IL-1β in the hypothalamus (Fig. 3c). The increased gene expression levels observed in the brain were accompanied by elevated GFAP immunoreactivity in both the CA1 and CA3 regions with no significant difference in Iba-1 immunoreactivity (Fig. 3d–g).

Next, we examined whether injections of IL-1β and TNF-α in the gums can modulate anxiety-like behavior and cognitive functions of mice. The % weight changes from baseline during the experimental period are shown in Additional file 1: Fig. S6. From the open field test, cytokines-injected mice displayed hyperactivity as shown by increased total distance traveled compared to control (Additional file 1: Fig. S7a). In addition, there was no difference in the % of time spent in the center between the groups (Additional file 1: Fig. S7b), suggesting that both groups did not exhibit anxiety-like behavior. From the spontaneous Y maze test, injections of cytokines did not significantly alter % spontaneous alternation in mice (Additional file 1: Fig. S7c) but marginally increased the number of total arm entries (Additional file 1: Fig. S7d). Finally, from the puzzle box test, mice injected with cytokines exhibited a significantly longer latency to reach the goal zone 1 min after the first exposure to the burrowing task (Additional file 1: Fig. S7e), suggesting an impairment of short-term memory function.

### Experimental periodontitis induced by heat-killed Pg potentiated pathological features and exacerbated cognitive impairment in 3 × Tg-AD mice

**Fig. 4:**
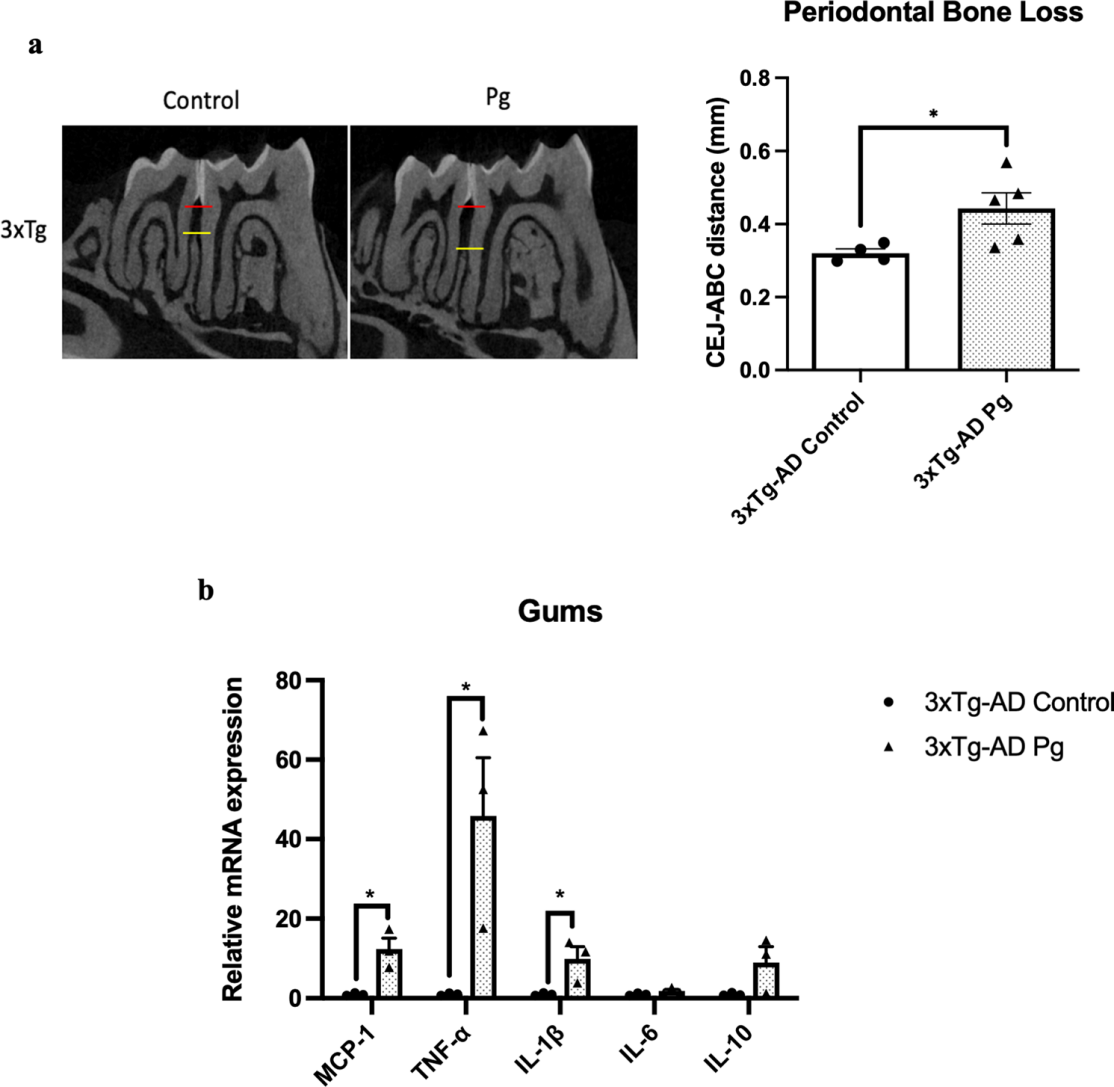

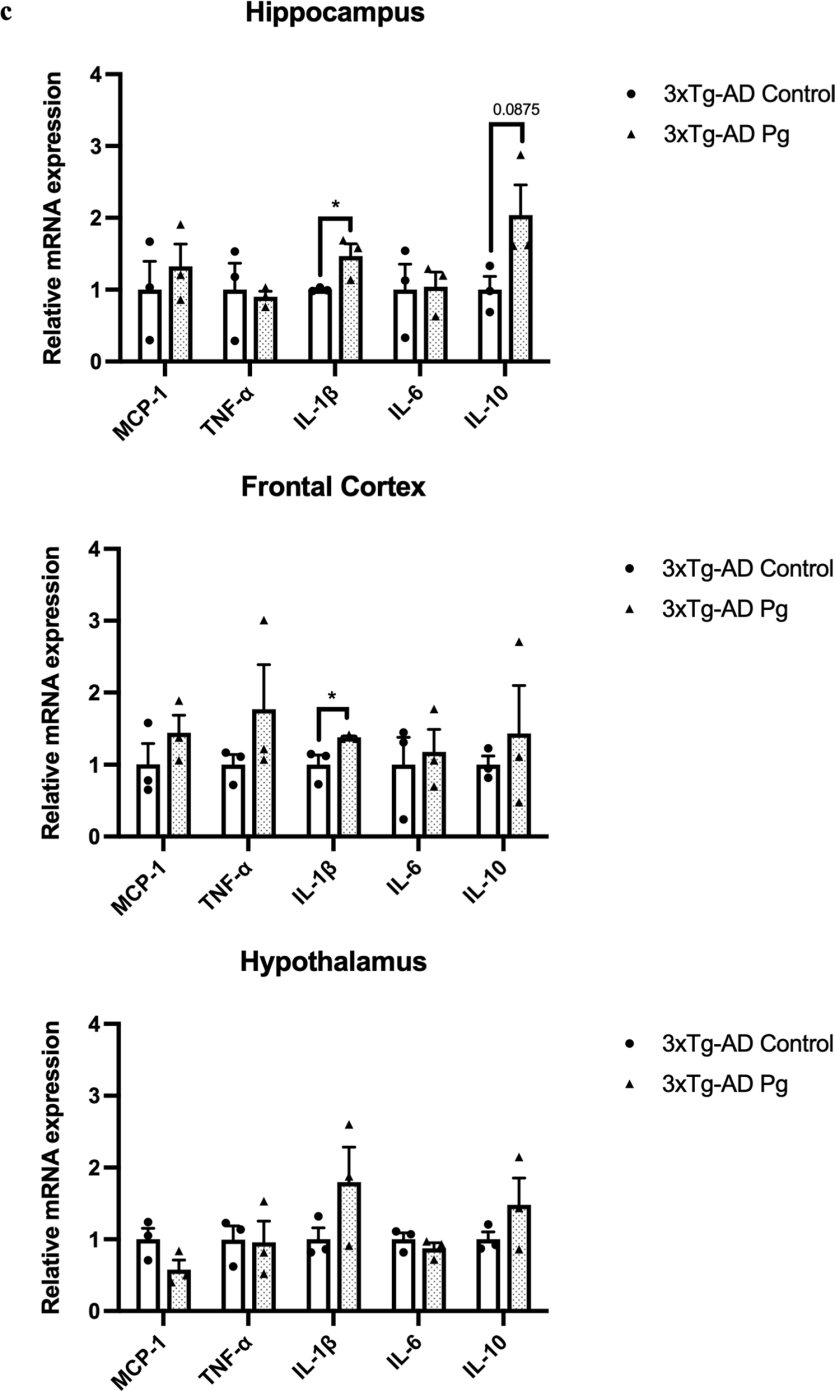

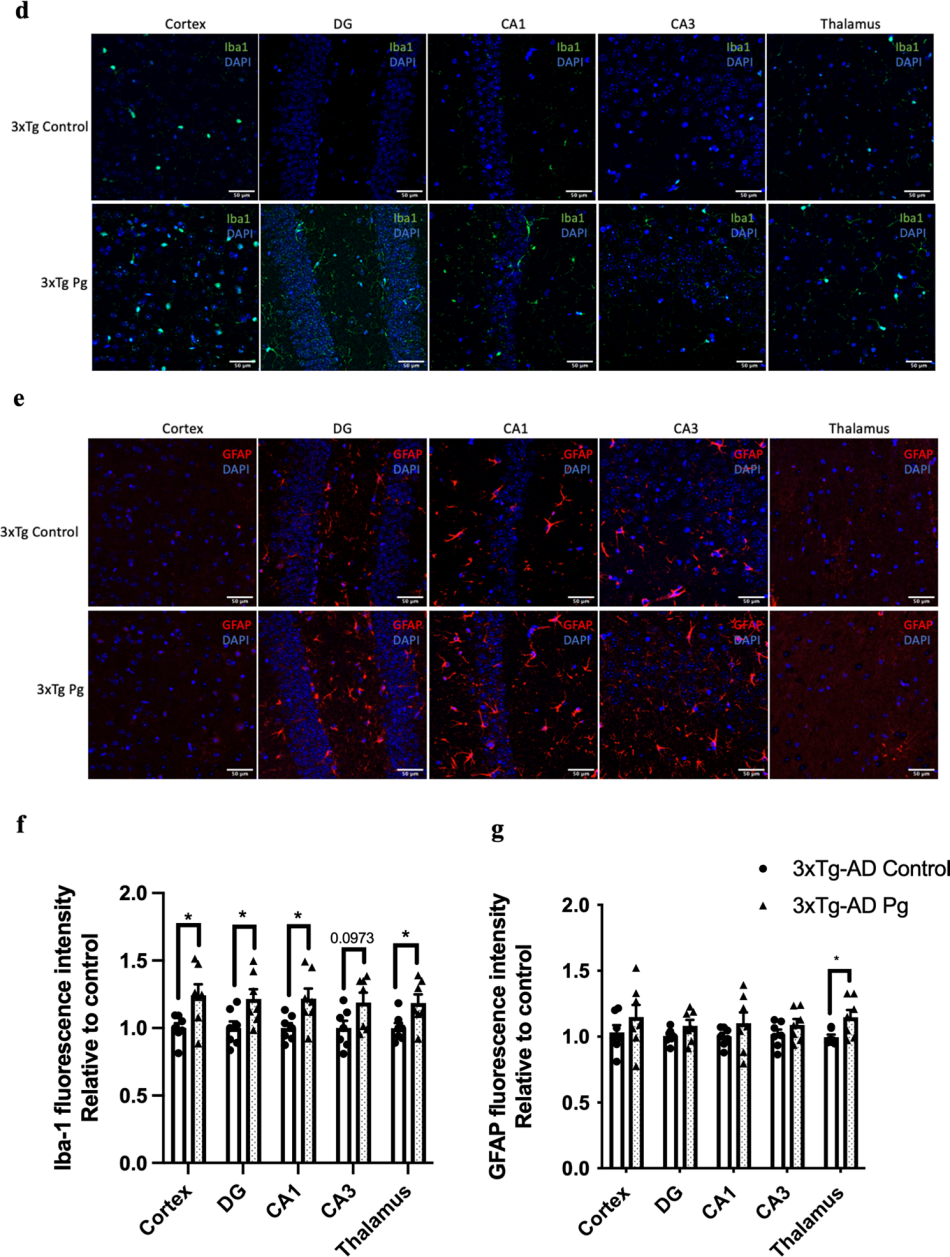
Bacterial-induced periodontitis increased immune responses in the gums and the brains of 3 × Tg-AD mice. **a** Representative two-dimensional sagittal images of the left mandibular jaws from 3 × Tg-AD mice in each group. Red and yellow bars indicate the distance from the cementoenamel junction (CEJ) to the alveolar bone crest (ABC) (Left); Quantitative measurements of the vertical bone loss (mm) from CEJ to ABC between the first and second molars (Right). Data are presented as mean ± SEM (*n* = 4–5). **b** Relative mRNA expression levels of inflammatory mediators in the gums of 3 × Tg-AD mice in each group. **c** Relative mRNA expression levels of inflammatory mediators in the hippocampus, frontal cortex, and hypothalamus of 3 × Tg-AD mice in each group. Data are presented as mean ± SEM (*n* = 3). **d** Representative images of immunofluorescence staining for Iba-1-positive microglia (green) and DAPI (blue) in the cortex, sub-regions of the hippocampus, and thalamus of 3 × Tg-AD mice in each group. **e** Representative images of immunofluorescence staining for GFAP-positive astrocytes (red) and DAPI (blue) in the cortex, sub-regions of the hippocampus, and thalamus of 3 × Tg-AD mice in each group. **f**, **g** Quantification of (**f**) Iba-1 and (**g**) GFAP immunofluorescence intensity in the cortex, sub-regions of the hippocampus, and thalamus of 3 × Tg-AD mice in each group. Data are presented as mean ± SEM (*n* = 7). Statistical analysis was done using unpaired *t* test. *p < 0.05

**Fig. 5:**
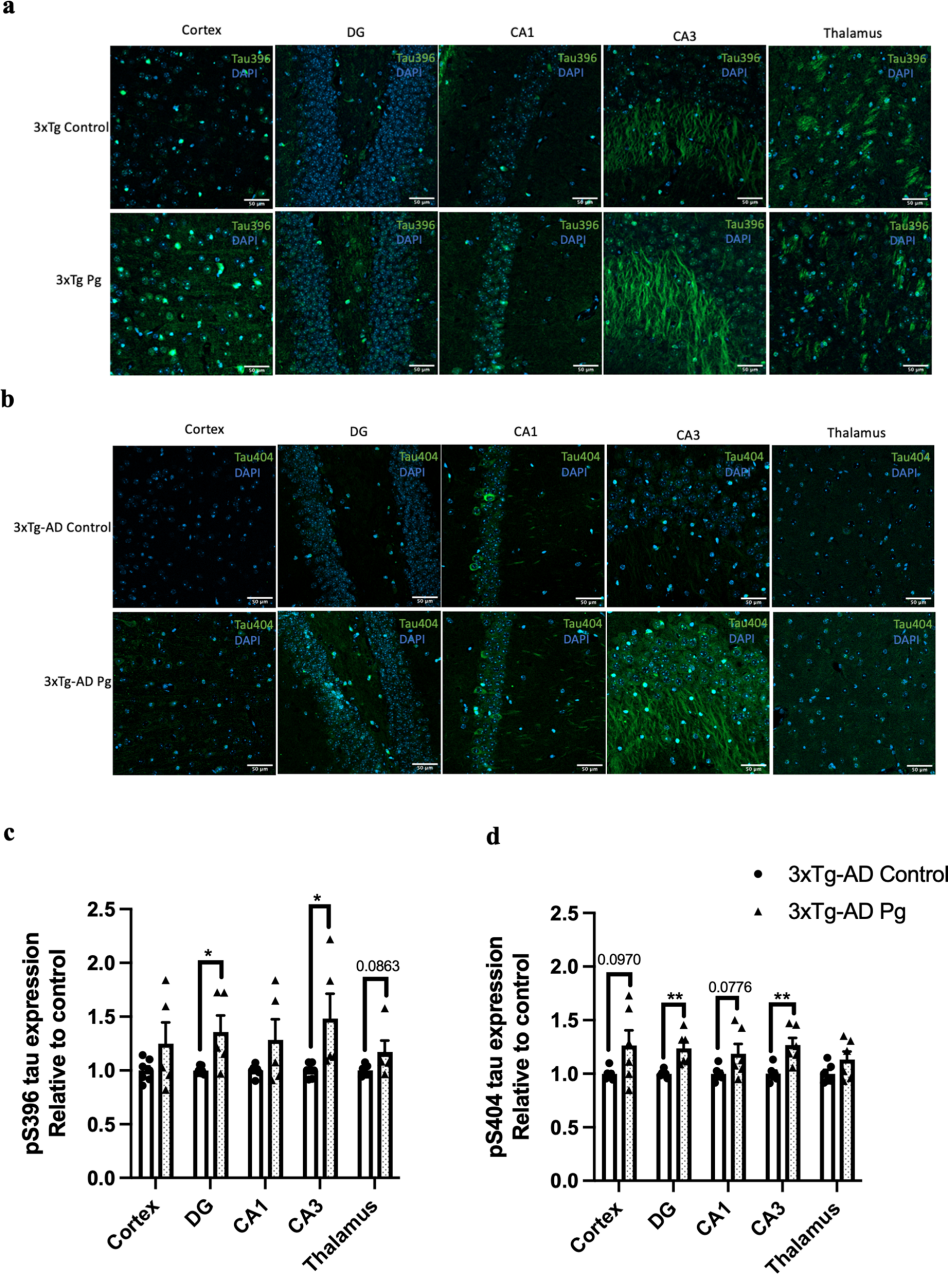
Bacterial-induced periodontitis increased phosphorylated tau proteins immunoreactivity in the brains of 3 × Tg-AD mice. **a** Representative images of immunofluorescence staining for phosphorylated tau Ser396 (green) and DAPI (blue) in the cortex, sub-regions of the hippocampus, and thalamus of 3 × Tg-AD mice in each group. **b** Representative images of immunofluorescence staining for phosphorylated tau Ser404 (green) and DAPI (blue) in the cortex, sub-regions of the hippocampus, and thalamus of 3 × Tg-AD mice in each group. **c**, **d** Quantification of (**c**) phosphorylated tau Ser396 and (**d**) phosphorylated tau Ser404 immunofluorescence intensity in the cortex, sub-regions of the hippocampus, and thalamus of 3 × Tg-AD mice in each group. Data are presented as mean ± SEM (*n* = 5–7). Statistical analysis was done using unpaired *t* test. *p < 0.05, **p < 0.01

Transgenic mice have been widely used to study many aspects of AD pathophysiology. Up to date, two studies have used transgenic mouse models of AD with age-dependent brain deposition and accumulation of extracellular plaques to examine the effects of periodontitis on AD pathology [24, 25]. However, the absence of neurofibrillary tangles in these AD models made it challenging to recapitulate all the pathological features of AD. We thus sought to employ a triple-transgenic mouse model of AD (3 × Tg-AD) to investigate whether chronic systemic inflammation, if any, caused by periodontitis may exacerbate pre-existing AD pathology. *Pg*-injected mice displayed significantly increased periodontal bone loss compared to control mice (Fig. 4a). Periodontitis induced by heat-killed *Pg* did not further increase the total bacterial load (Additional file 1: Fig. S8) but led to increased gene expression levels of MCP-1, TNF-α, and IL-1β in the gums of AD mice (Fig. 4b) and CRP levels in the plasma (Additional file 1: Fig. S2b). Elevated gene expression levels of IL-1β were also seen in both the hippocampus and the frontal cortex regions of AD mice with periodontitis (Fig. 4c). *Pg*-injected AD mice also exhibited increased Iba-1 and GFAP immunoreactivities in the brains (Fig. 4d-g). We next determined whether periodontitis could lead to the exacerbation of tau and Aβ pathology. Significantly greater immunoreactivities of phosphorylated tau396 and tau404 in the dentate gyrus and CA3 regions were detected in *Pg*-injected AD mice (Fig. 5a–d). A significantly greater % of 6E10-immunoreactive area was also observed in the cortex and CA1 regions of *Pg*-injected AD mice (Additional file 1: Fig. S9a, c).

To examine the effects of periodontitis induced by *Pg* on general exploratory and anxiety behavior in AD mice, the open field test was performed after five weeks of repeated injections. The % weight changes from baseline during the experimental period are shown in Additional file 1: Fig. S10. No significant differences in the total distance traveled and the % of time spent in the center between the groups were observed (Additional file 1: Fig. S11a, b). Findings from the spontaneous Y maze test further revealed significantly reduced % spontaneous alternation in infected mice compared to control, with no difference in the number of total arm entries (Additional file 1: Fig. S11c, d). This indicates that periodontitis induced by *Pg* exacerbated impairment of short-term memory in AD mice. When assessing general cognition and executive functions using the puzzle box test, infected mice took a significantly longer time to reach the goal zone 24 h after the first exposure to the burrowing task (Additional file 1: Fig. S11e). This suggests that periodontitis induced by *Pg* exacerbated impairment of long-term memory in AD mice.

### Ligature-induced periodontitis potentiated pathological features and exacerbated cognitive impairment in 3 × Tg-AD mice

**Fig. 6:**
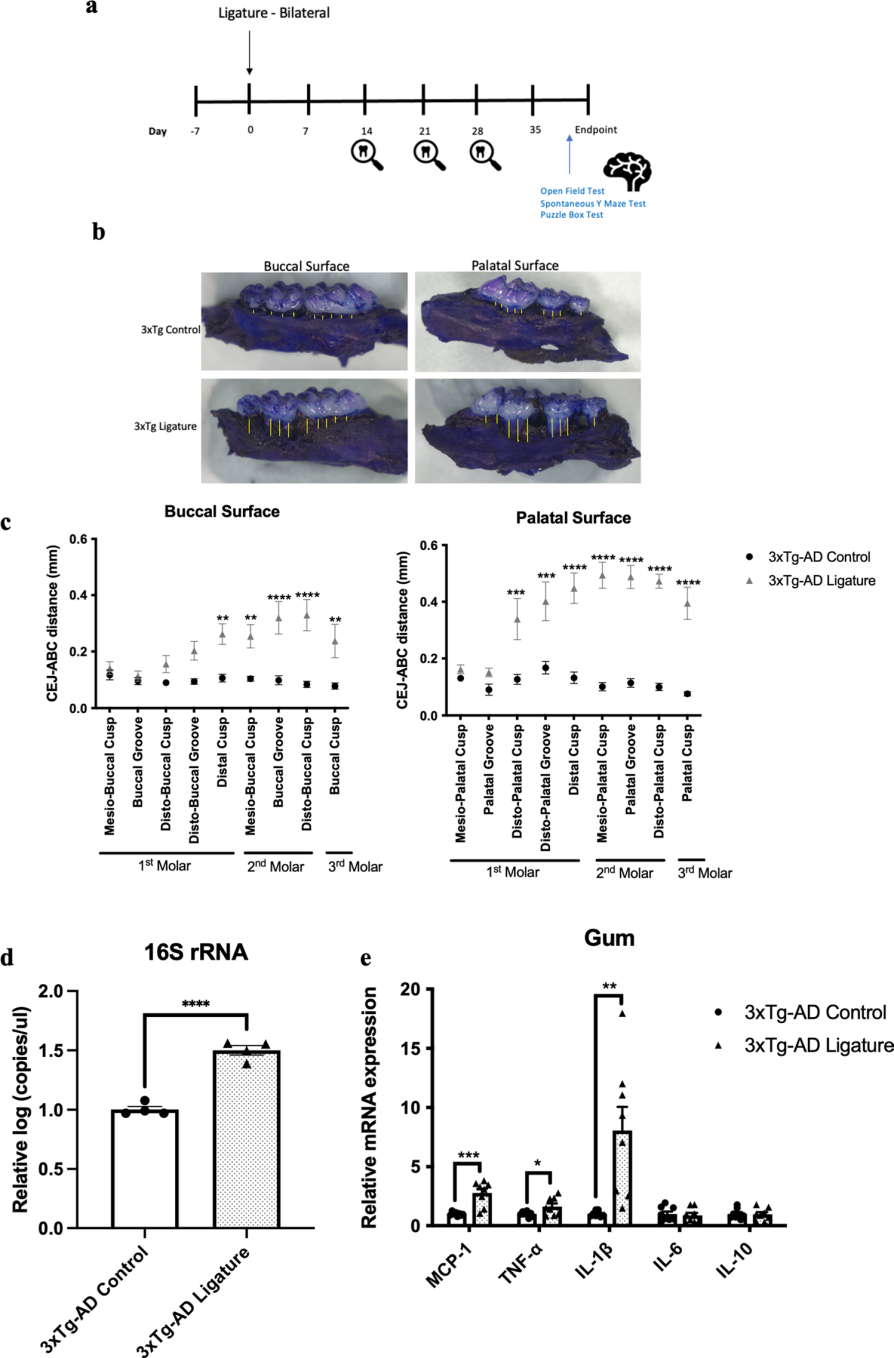

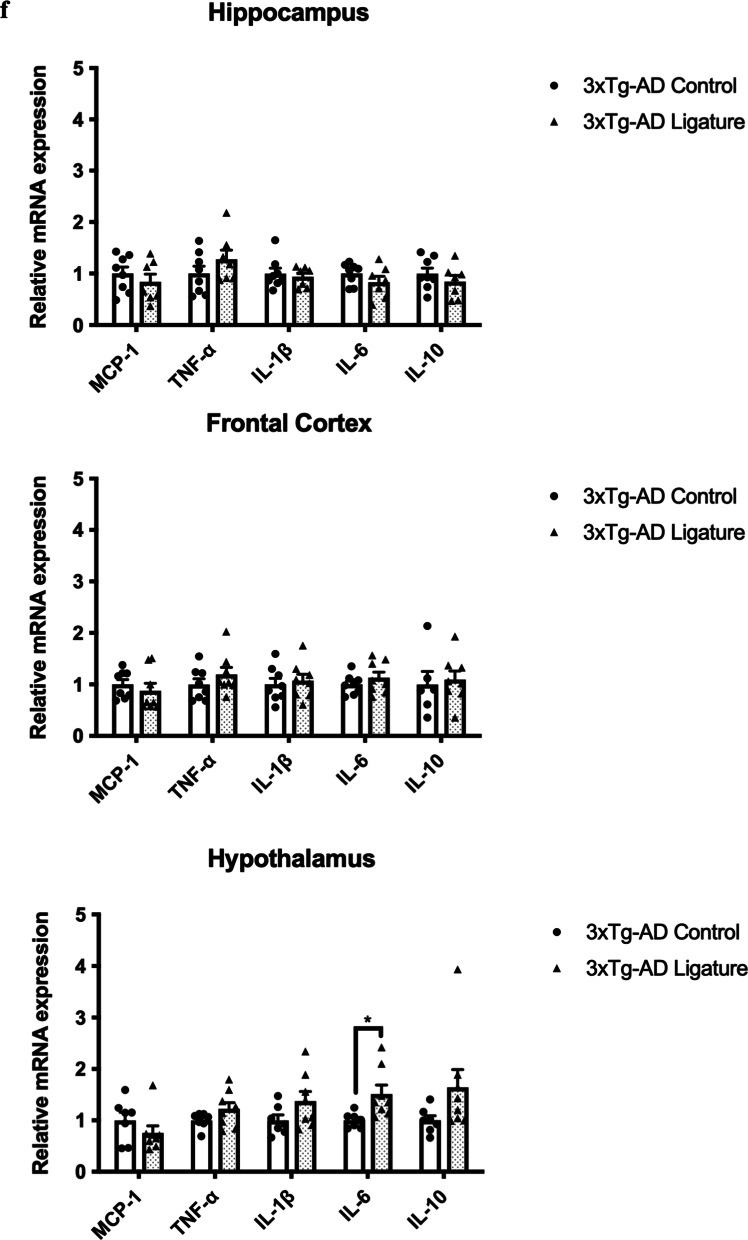

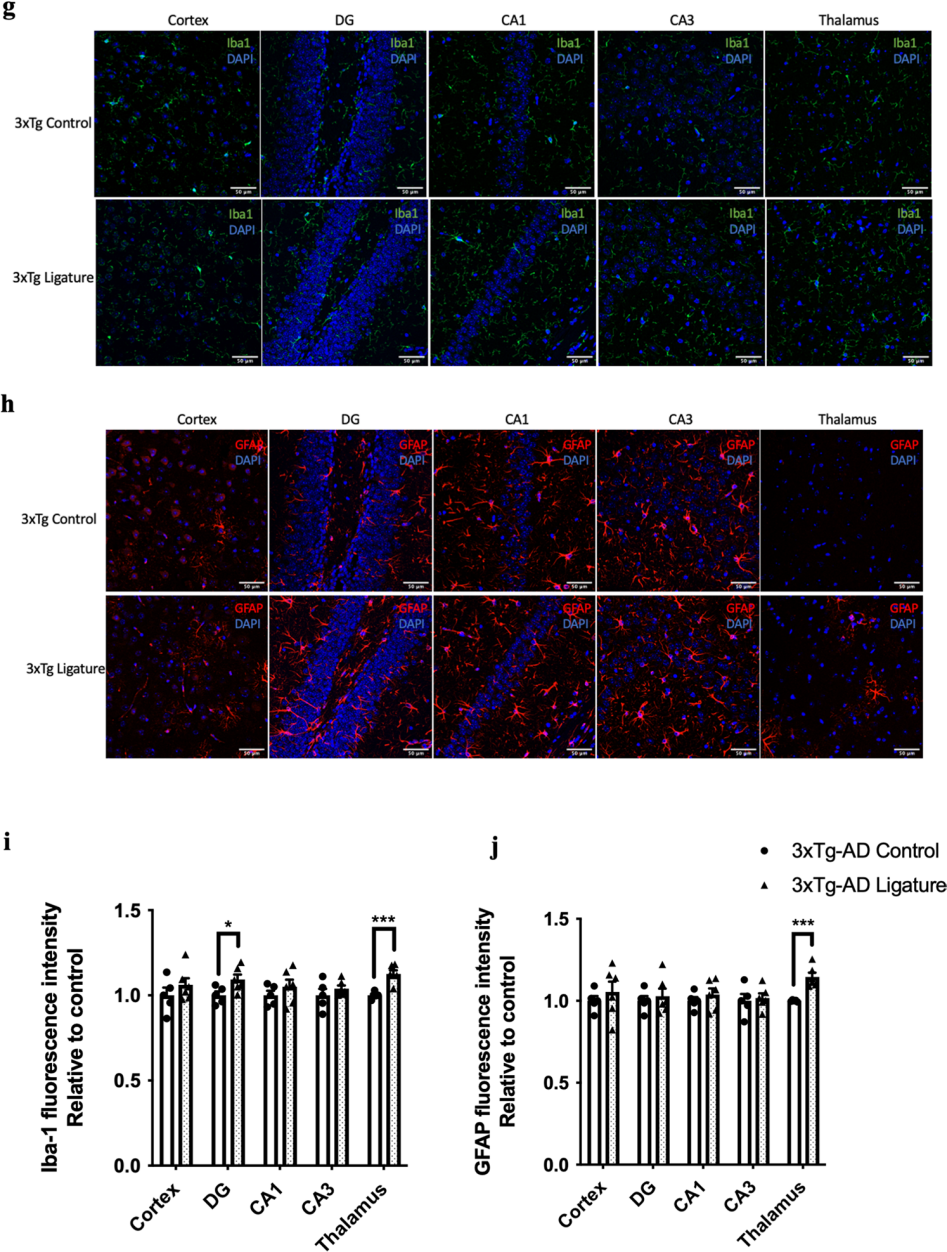
Ligature-induced periodontitis increased immune responses in the gums and the brains of 3 × Tg-AD mice. **a** Schematic illustration of the experimental timeline. **b** Representative images of the stained maxillary jaws from 3 × Tg-AD mice in each group viewed at both buccal and palatal sides. Yellow bars represent the distance from the cementoenamel junction (CEJ) to the alveolar bone crest (ABC). **c** Quantitate measurements of the vertical bone loss (mm) from CEJ to ABC at both buccal and palatal sides. Data are presented as mean ± SEM (*n* = 5). Statistical analysis was done using two-way ANOVA with Tukey’s post-hoc test. **d** The total bacterial load detectable within gum tissues was measured by 16S rRNA real-time PCR at 5 weeks post-ligation. Data are present as mean ± SEM (*n* = 4). **e** Relative mRNA expression levels of inflammatory mediators in the gums of 3 × Tg-AD mice in each group. **f** Relative mRNA expression levels of inflammatory mediators in the hippocampus, frontal cortex, and hypothalamus of 3 × Tg-AD mice in each group. Data are presented as mean ± SEM (*n* = 7–8). **g** Representative images of immunofluorescence staining for Iba-1-positive microglia (green) and DAPI (blue) in the cortex, sub-regions of the hippocampus, and thalamus of 3 × Tg-AD mice in each group. **h** Representative images of immunofluorescence staining for GFAP-positive astrocytes (red) and DAPI (blue) in the cortex, sub-regions of the hippocampus, and thalamus of 3 × Tg-AD mice in each group. **i**, **j** Quantification of (**i**) Iba-1 and (**j**) GFAP immunofluorescence intensity in the cortex, sub-regions of the hippocampus, and thalamus of 3 × Tg-AD mice in each group. Data are presented as mean ± SEM (*n* = 5–6). Statistical analysis was done using unpaired *t* test. *p < 0.05, **p < 0.01, ***p < 0.001, ****p < 0.0001

**Fig. 7:**
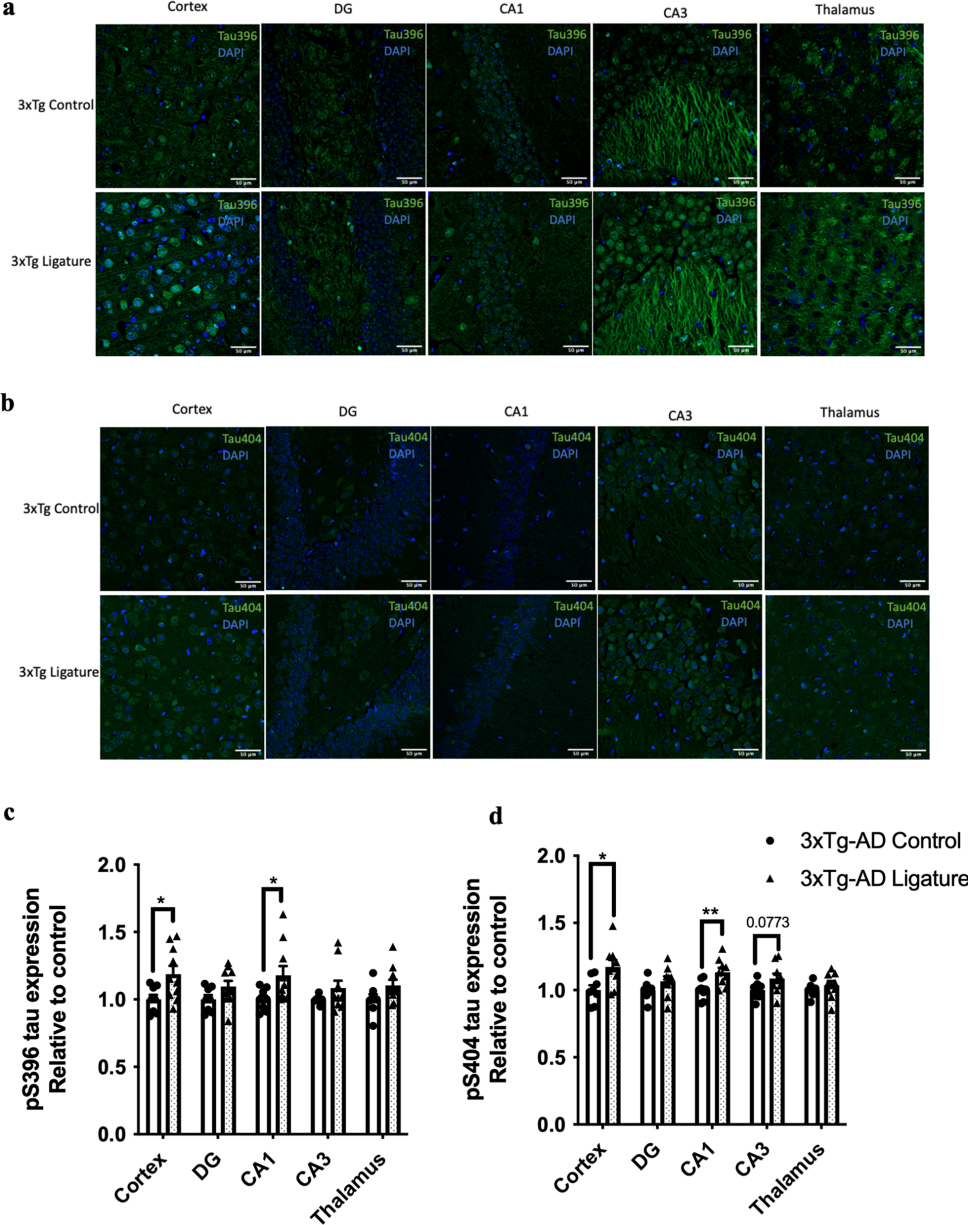
Ligature-induced periodontitis increased phosphorylated tau proteins immunoreactivity in the brains of 3 × Tg-AD mice **a** Representative images of immunofluorescence staining for phosphorylated tau Ser396 (green) and DAPI (blue) in the cortex, sub-regions of the hippocampus, and thalamus of 3 × Tg-AD mice in each group. **b** Representative images of immunofluorescence staining for phosphorylated tau Ser404 (green) and DAPI (blue) in the cortex, sub-regions of the hippocampus, and thalamus of 3 × Tg-AD mice in each group. **c**, **d** Quantification of (**c**) phosphorylated tau Ser396 and (**d**) phosphorylated tau Ser404 immunofluorescence intensity in the cortex, sub-regions of the hippocampus, and thalamus of 3 × Tg-AD mice in each group. Data are presented as mean ± SEM (*n* = 8–10). Statistical analysis was done using unpaired *t* test. *p < 0.05, **p < 0.01

As periodontitis induced by the injection of heat-killed *Pg* did not increase the total bacterial load in the gums of mice, we sought to evaluate another model of periodontitis using silk ligatures. The timeline of the study is shown in Fig. 6a. Ligature-induced periodontitis significantly increased periodontal bone loss in 3 × Tg-AD (Fig. 6b, c), which was accompanied by increased total bacterial load (Fig. 6d) and elevated gene expression levels of MCP-1, TNF-α and IL-1β in the gums (Fig. 6e). When assessing various inflammatory cytokines in different brain regions by real-time PCR, significantly increased IL-6 was detected in the hypothalamus of ligated 3 × Tg-AD mice compared to control (Fig. 6f). Ligature-induced periodontitis also increased Iba-1 immunoreactivity in the DG and thalamus regions as well as GFAP immunoreactivity in the thalamus region of 3 × Tg-AD mice (Fig. 6g–j). Besides that, significantly greater immunoreactivities of phosphorylated tau396 and tau404 in the cortex and CA1 regions were detected in 3 × Tg-AD mice with ligature placement (Fig. 7a–d). In addition, a significantly greater % 6E10-immunoreactive area was observed in the cortex and CA1 regions of 3 × Tg-AD mice with ligature placement (Additional file 1: Fig. S9b, d).

The % weight changes from baseline during the experimental period are shown in Additional file 1: Fig. S12. A significant reduction in the % weight from baseline was observed after ligature placement in the first week. However, all mice started to regain weight after week one and their weights remained stable until the experimental endpoint. Results from the open field test revealed that ligature-induced periodontitis did not alter exploratory behavior in 3 × Tg-AD mice and there was no difference in the % of time spent in the center (Additional file 1: Fig. S13a, b). In addition, ligature-induced periodontitis significantly reduced % spontaneous alternation in 3 × Tg-AD mice compared to control, with no difference in the number of total arm entries (Additional file 1: Fig. S13c, d). This indicates that periodontitis induced by ligature placement exacerbated the impairment of short-term memory in AD mice. Furthermore, when assessing problem-solving ability and executive functions using the puzzle box test, 3 × Tg-AD with ligature placement exhibited a significantly longer latency to reach the goal zone 24 h after the first exposure to the burrowing task (Additional file 1: Fig. S13e). This suggests that periodontitis induced by ligature placement also exacerbated the impairment of long-term memory in AD mice.

### Injections of IL-1β and TNF-α resulted in the loss of TH-immunoreactivity and increased microglial immunoreactivity in the locus coeruleus of mice

As degeneration of the locus coeruleus (LC) occurs very early in the pathogenesis of AD, we also sought to investigate the potential impact of peripheral inflammatory mediators on LC integrity. To do so, double-immunolabeling of LC sections using tyrosine hydroxylase (TH), a marker for norepinephrine neurons with Iba-1 (Sigma Aldrich), and TH with GFAP were performed in the LC region of 3 × Tg-AD mice injected with and without mixed cytokines. Here, we observed increased Iba-1 immunoreactivity and decreased TH fluorescence intensity in the LC of mice injected with IL-1β and TNF-α in the gums (Additional file 1: Fig. S14), both of which are indicative of LC neuronal damage and inflammation.

### Ligature-induced periodontitis resulted in decreased MT ratios in the brain of 3 × Tg-AD mice

**Fig. 8:**
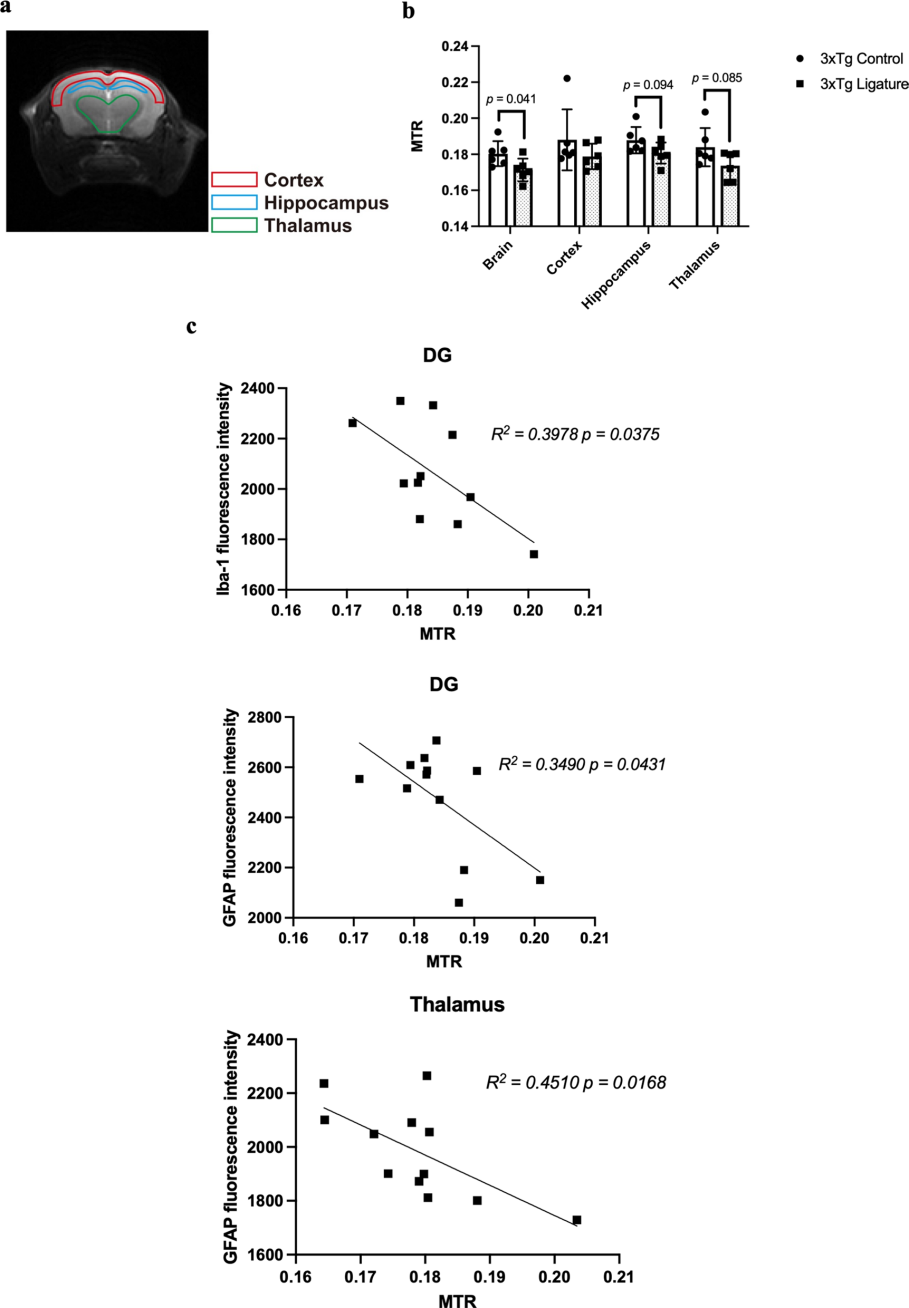
Comparison of MT ratios between control and ligated 3 × Tg-AD mice. **a** T2-weight image depicting the different regions of interest in the coronal brain slices for the cortex, hippocampus, and thalamus. **b** Group comparison of MT ratios in the cortex, hippocampus, and thalamus between control and ligated 3 × Tg-AD mice. **c** Linear regression analysis of the correlation between MT ratios and Iba-1/GFAP immunoreactivity. Data are presented as mean ± SEM (*n* = 5–6). Statistical analysis was done using unpaired *t* test

MT is an MRI contrast used to assess changes in macromolecules in the brain [26], including dementia and AD [27–29]. Here, we also determined whether the neuropathological changes observed in 3 × Tg-AD mice with periodontitis could be detected using MT contrast. We noted a decreased MT ratio in the brain, particularly in the hippocampus and the thalamus region of 3 × Tg-AD mice with periodontitis compared to control mice (Fig. 8a, b). The observed changes in MT ratio also correlated significantly to Iba-1 immunoreactivity in the DG and GFAP immunoreactivity in the DG and thalamus region (Fig. 8c), indicating that periodontitis-induced glial responses may be responsible for the decreased MT ratio observed in 3 × Tg-AD mice.

## Discussion

Over the last decade, there has been growing evidence supporting the role of periodontal pathogens and systemic inflammation in AD pathogenesis [30]. Our present study demonstrated that increased cytokine immune responses in the periodontium contributed to the risk of AD in nTg mice and exacerbated neuropathology and cognitive impairment in 3 × Tg-AD mice.

Here, we initially investigated the effects of aging on the periodontium of C57BL/6 J and 3 × Tg-AD mice. With aging, significantly reduced % remaining bone and increased MCP-1, IL-1β, and TNF-α gene expression levels in the gums were observed. Indeed, aging causes alterations in the immune system, leading to dysregulated immune responses [31, 32]. These findings were in concordance with previous studies reporting that in the elderly, moderate loss of alveolar bone and elevated gingival levels of pro-inflammatory cytokines can often be observed [32, 33]. Several studies utilizing different animal models have also demonstrated age-associated changes in periodontal bone levels and cytokine expression levels in the gingiva [34, 35].

To date, the oral cavity housed approximately 1000 bacterial species [36]. Among them, *Pg* is a gram-negative anaerobic bacterium identified to be the major periodontopathic/dysbiotic species [16]. Recent findings have provided evidence that *Pg* and its virulence factors could access the brains contributing to the pathogenesis of AD directly [37]. The presence of antibodies against *Pg* has also been found to be associated with increased levels of TNF-α in the systemic circulation of AD patients [38]. In addition, chronic oral administration of *Pg* resulted in increased levels of pro-inflammatory cytokines and the formation of extracellular plaques in the brains of young C57BL/6 mice [39]. Several lines of evidence have reported that the use of heat-killed *Pg* mimics the effects of using *Pg* LPS [40–42]. Certainly, the heating of the whole cells can modify or alter the protective protein epitopes of *Pg* and permit the presentation of heat-stable polysaccharides of the bacterium capsule or the O-polysaccharide of the LPS, triggering immune responses [42]. Accumulating data further shows that *Pg* has evolved several mechanisms to evade the host immune system. Of note, the heat-instable gingipains have been shown to possess catalytic activities targeting a wide array of cytokines and chemokines [43, 44]. Taken together, these indicate that heat-killed *Pg* can elicit a higher immune response than viable bacteria, providing a rationale for our decision in using heat-killed *Pg* as a model to stimulate the host immune response.

One major limitation to the induction of experimental periodontitis using heat-killed *Pg* is that the model relied heavily on repeatedly stimulating the host inflammatory immune response to induce systemic inflammation and thus could not fully recapitulate the full pathogenesis of human periodontitis. However, injections of heat-killed *Pg* in nTg mice led to gum inflammation as shown by increased gene expression levels of IL-1β and TNF-α in the gums as well as systemic inflammation as shown by elevated CRP levels in the plasma. The increased systemic immune responses observed were concomitant with increased neuroimmune responses and tau phosphorylation. These changes were also accompanied by deficits in long-term memory. Altogether, these findings from nTg support that systemic inflammation resulting from periodontitis contributes to the development of AD tau pathology and subsequently leads to cognitive decline.

As IL-1β and TNF-α were found to be of high importance concerning the conversion of mild cognitive impairment to dementia [13], we next sought to evaluate whether the direct injections of these two cytokines into the gums of nTg mice could contribute to the risk of AD onset through modulating neuroimmune responses. Injections of IL-1β and TNF-α in the gums resulted in periodontal bone loss. This is expected as these two cytokines are known to be the major mediators of periodontal bone destruction in the pathogenesis of periodontitis [45]. Elevated IL-1β and TNF-α gene expression levels, as well as increased astrocytic immunoreactivity, were also observed in the hippocampus of mice injected with mixed cytokines. Even though we cannot eliminate the possibility of systemic redistribution of the injected cytokines, the observation that it led to significantly increased gene expression of both cytokines in the brain after mandibular buccal vestibule injections further demonstrates the association between periodontal inflammation and neuroimmune responses. Although this research provides convincing evidence for the importance of inflammatory mediators in connecting periodontitis to AD, the mechanistic link between the two diseases is still lacking. Hence, additional research involving the use of gene knock-out mice or specific cytokine inhibitors would further our mechanistic understanding of the relationship between periodontitis and AD.

Periodontitis is highly prevalent in the elderly population, especially for those with cognitive impairment [46]. To the best of our knowledge, the effects of experimental periodontitis on cognitive function and neuropathology of 3 × Tg-AD mice have not been previously reported. One study performed oral inoculation of *Pg* in amyloid precursor protein (APP) transgenic mice and noted higher levels of pro-inflammatory cytokines and deposition of amyloid plaques in the brain along with a reduction in cognitive function compared to the control group [24]. More recently, another group induced periodontitis using the ligature method in 5 × FAD mice reported increased insoluble Aβ42 and unresolved inflammation in the brain [25]. Both studies employed AD transgenic mice that recapitulate amyloid pathology as that seen in AD. However, as AD is also characterized by the presence of neurofibrillary tangles, 3 × Tg-AD mice bearing mutations on presenilin 1, APP, and tau protein were employed in this study to investigate the impacts of periodontitis on AD.

In concordance with findings from heat-killed *Pg-*infected nTg mice, injections of heat-killed *Pg* in 3 × Tg-AD mice also led to gum inflammation as well as systemic inflammation as shown by elevated CRP levels in the plasma. The role of systemic inflammation on glial responses in AD was further demonstrated with significantly increased Iba-1 and GFAP immunoreactivities in the brains of infected 3 × Tg-AD mice. Tau phosphorylation of serine resides 396 and 404 occurs in the early stages of AD. 3 × Tg-AD mice infected with heat-killed *Pg* displayed significantly increased tau396 and 404 immunoreactivities in the DG and CA3 regions of the hippocampus. Apart from the injection of heat-killed *Pg*, there are various animal models of periodontitis in periodontal research. Among them, ligature-induced periodontitis is a widely used model that best reflects human periodontitis as it promotes the accumulation of oral bacteria leading to inflammation and subsequently periodontal bone destruction. Although our second model involving the ligation of second maxillary molars best reflects human periodontitis, we cannot rule out the possibility of any mechanical injury caused by ligature placement, which could thus aggravate periodontal tissue destruction and contribute to bone loss. However, efforts were made to minimize any damage to the periodontium during surgery. We found that ligature placement led to significantly increased total bacterial load in the recovered sutures as well as those detectable in homogenized gum tissues. Several studies have demonstrated that the oral microbiome may modulate brain functions [37, 47, 48]. Here, we did not find a significant increase in the total bacterial load in the brains of 3 × Tg-AD mice at 7 days and 5 weeks following ligature placement (Additional file 1: Fig. S15), indicating that the observed pathological changes in 3 × Tg-AD mice are perhaps due to the consequences of elevated cytokine immune responses in the gums. Given that 3 × Tg-AD mice also exhibit Aβ pathology, we further demonstrated increased 6E10 immunoreactivity in the cortex and CA1 regions of heat-killed *Pg–*infected 3 × Tg-AD mice and the CA1 region of ligated 3 × Tg-AD mice. Concomitant with these neuropathological changes, our findings also showed that periodontitis induced by *Pg* and ligature placement was associated with exacerbated cognitive dysfunctions of 3 × Tg-AD mice.

When assessing the transfer of magnetization using a conventional MT contrast experiment, decreased MT ratios were noted in the brain of 3 × Tg-AD mice with periodontitis, which correlated well with gliosis in the DG and the thalamus region. Our interpretation of a reduced MT ratio due to heightened glial responses is further supported by histological studies in which ligature-induced periodontitis led to significant increases in Iba-1 immunoreactivity in the DG and thalamus regions, and GFAP immunoreactivity in the thalamus region of 3 × Tg-AD mice after ligature placement. This finding is also in concordance with another study that observed a lower MT ratio in the hypothalamus of 6 months old and the thalamus of 8 months old APP/PS1 mice compared to WT mice. In their study, the lower MT ratios correlated well with gliosis [49]. Similarly, the extent of astrogliosis was also found to correlate with decreased MT ratios in a rat model of traumatic brain injury [50]. Previous clinical studies have also reported reduced MT ratios in mild cognitive impairment and AD patients [28, 51]. In summary, we attribute our findings to increased glial responses as a result of systemic inflammation induced by periodontitis in 3 × Tg-AD mice, which further strengthen the role of inflammation in connecting periodontitis to AD.

Despite the significance of peripheral inflammatory cytokines in mediating neuroimmune responses and modulating cognitive functions, the mechanism linking cytokine immune responses from the periodontium to the brain remains to be established. We hypothesize that the neuropathological events resulting from periodontal inflammation are likely due to the dysfunction of the LC system. LC, a small nucleus that resides in the brainstem, is the primary source of norepinephrine in the CNS and has extensive projections to numerous regions of the forebrain and spinal cord to regulate diverse physiological functions including cognitive functions and neuroimmune responses [52]. It is well-documented that LC cell numbers and brain norepinephrine levels are reduced during the aging process, and, to a greater extent, in AD [53]. Goto et al. demonstrated that tooth loss in 3 × Tg-AD mice led to LC damage and subsequently hippocampal neuronal degeneration [54]. Here, we observed increased Iba-1 immunoreactivity and decreased TH fluorescence intensity in the LC of 3 × Tg-AD mice injected with IL-1β and TNF-α in the gums, which further provided evidence for the presence of LC neuronal damage and inflammation induced by increased cytokine immune responses in the gums. In addition, many studies have highlighted the dysregulation of the LC system in the development of neuroinflammation and cognitive dysfunction in AD [55–58]. Loss of LC neurons has also been associated with increased amyloid burden and tau pathology in AD cases [59, 60]. In this present study, the presence of LC stress/damage and inflammation could thus account for the exacerbated neuropathology and cognitive impairment in 3 × Tg-AD mice with periodontitis. Taken together, these data may warrant a hypothesis whereby the LC-norepinephrine pathway is involved in the association between periodontitis and AD.

## Conclusions

To conclude, systemic inflammation induced by experimental periodontitis modulated neuroimmune responses, tau phosphorylation, as well as behavior and cognition, contributing to the risk of AD. It also potentiated AD pathological features and exacerbated cognitive impairment in 3 × Tg-AD mice. Collectively, these findings provide a strong indication of the potential role of IL-1β and TNF-α in modulating the risk of both AD and periodontitis.

## Supplementary Information

**Additional file 1: Table S1.** List of primer sequences used in this study. **Fig. S1.** Assessment of bone mineral density from the right mandibular jaws of C57BL/6J and 3×Tg-AD mice at 3 months, 8 months, and 15-16 months of age. (a) A representative two-dimensional sagittal image of a right mandibular jaw depicting the region of interest (highlighted in yellow) for the quantitative measurement of bone mineral density. (b) Quantitative analysis of bone mineral density. Data are presented as mean ± SEM (C57BL/6J mice: *n* = 5/age group, 3×Tg-AD mice: *n* = 9-11/age group). Statistical analysis was done using one-way ANOVA with Tukey’s post-hoc test. ***p < 0.001. **Fig. S2.** Periodontitis induced by the injection of heat-killed Porphyromonas gingivalis resulted in elevated CRP levels in the plasma. (a) Plasma CRP concentration in nTg mice from each group. (b) Plasma CRP concentration in 3×Tg-AD mice from each group. Data are presented as mean ± SEM (*n* = 5-7). Statistical analysis was done using unpaired *t* test. *p < 0.05. **Fig. S3.** Periodontitis induced by the injection of heat-killed Porphyromonas gingivalis resulted in increased phosphorylated tau proteins immunoreactivity in the brains of non-transgenic (nTg) mice. (a) Representative images of immunofluorescence staining for phosphorylated tau Ser396 (green) and DAPI (blue) in the cortex, sub-regions of the hippocampus, and thalamus of nTg mice in each group. (b) Representative images of immunofluorescence staining for phosphorylated tau Ser404 (green) and DAPI (blue) in the cortex, sub-regions of the hippocampus, and thalamus of nTg mice in each group. (c, d) Quantification of (c) phosphorylated tau Ser396 and (d) phosphorylated tau Ser404 immunofluorescence intensity in the cortex, sub-regions of the hippocampus, and thalamus of nTg mice in each group. Data are presented as mean ± SEM (*n* = 6). Statistical analysis was done using unpaired *t* test. *p < 0.05, **p < 0.01. **Fig. S4.** Non-transgenic (nTg) mice body weight expressed as a percent change from baseline during the experimental period of heat-killed Porphyromonas gingivalis injections. Data are presented as mean ± SEM (*n* = 7-8). Statistical analysis was done using two-way ANOVA with Bonferroni’s post-hoc test. **Fig. S5.** Periodontitis induced by the injection of heat-killed Pg resulted in impairment of long-term memory in non-transgenic (nTg) mice. (a, b) Open field test for the assessment of general exploration and anxiety behavior. (a) Total distance traveled and (b) the percentage of time spent in center were examined. (c, d) Spontaneous Y maze test for the assessment of short-term spatial working memory. (c) Percentage spontaneous alternation and (d) the number of total arm entries were examined. (e) Puzzle box test for the assessment of problem-solving abilities and executive functions. Data are presented as mean ± SEM (*n* = 12-14). Statistical analysis was done using unpaired *t* test. **p < 0.01. **Fig. S6.** Non-transgenic (nTg) mice body weight expressed as a percent change from baseline during transgenic (nTg) mice body weight expressed as a percent change from baseline during transgenic (nTg) mice body weight expressed as a percent change from baseline during the experimental period of mixed cytokines injections. **Fig. S7.** Injections of mixed cytokines resulted in hyperactivity and impairment of short-term memory in non-transgenic (nTg) mice. (a, b) Open field test for the assessment of general exploration and anxiety behavior. (a) Total distance traveled and (b) the percentage of time spent in center were examined. (c, d) Spontaneous Y maze test for the assessment of short-term spatial working memory. (c) Percentage spontaneous alternation and (d) the number of total arm entries were examined. (e) Puzzle box test for the assessment of problem-solving abilities and executive functions. Data are presented as mean ± SEM (*n* = 7). Statistical analysis was done using unpaired *t* test. *p < 0.05, **p < 0.01. **Fig. S8.** Total bacterial load in the gums of 3×Tg-AD mice measured by 16S rRNA real-time PCR at the end of the experimental period. Tissue DNA spiked with 100 ng of Pg DNA was included as positive controls. Data are presented as mean ± SEM (*n* = 12). Statistical analysis was done using one-way ANOVA with Tukey’s post-hoc test. *p < 0.05. **Fig. S9.** Periodontitis induced by injection of heat-killed Pg as well as ligature placement resulted in increased 6E10 immunoreactivity in the brains of 3×Tg-AD mice. (a-b) Representative images of immunofluorescence staining for 6E10 (red) and DAPI (blue) in the cortex and sub-regions of hippocampus of 3×Tg-AD mice with and without (a) Pg injections and (b) ligature placement. (c-d) Quantification of 6E10 immunofluorescence intensity in the cortex and sub-regions of the hippocampus of 3×Tg-AD mice with and without (c) Pg injections and (d) ligature placement. Data are presented as mean ± SEM (*n* = 6-7). Statistical analysis was done using unpaired *t* test. *p < 0.05, **p < 0.01. **Fig. S10.** 3×Tg 3×Tg-AD mice body weight expressed as a percent change from baseline during the experimental period of heat-killed Pg injections. **Fig. S11.** Periodontitis induced by the injection of heat-killed Porphyromonas gingivalis resulted in impairment of both short- and long-term memory in 3×Tg-AD mice. (a, b) Open field test for the assessment of general exploration and anxiety behavior. (a) Total distance traveled and (b) the percentage of time spent in center were examined. Data are presented as mean ± SEM (*n* = 15-16). (c, d) Spontaneous Y maze test for the assessment of short-term spatial working memory. (c) Percentage spontaneous alternation and (d) the number of total arm entries were examined. Data are presented as mean ± SEM (*n* = 11-12). (e) Puzzle box test for the assessment of problem-solving abilities and executive functions. Data are presented as mean ± SEM (*n* = 12-13). Statistical analysis was done using unpaired *t* test. *p < 0.05, **p < 0.01. **Fig. S12**. 3×Tg-AD mice body weight expressed as a percent change from baseline during the experimental period of ligature placement. Data are presented as mean ± SEM (*n* = 12). Statistical analysis was done using two-way ANOVA with Bonferroni’s post-hoc test. **p < 0.01. **Fig. S13.** Ligature-induced periodontitis resulted in impairment of both short- and long-term memory in 3×Tg-AD mice. (a, b) Open field test for the assessment of general exploration and anxiety behavior. (a) Total distance traveled and (b) the percentage of time spent in center were examined. Data are presented as mean ± SEM (*n* = 17-19). (c, d) Spontaneous Y maze test for the assessment of short-term spatial working memory. (c) Percentage spontaneous alternation and (d) the number of total arm entries were examined. Data are presented as mean ± SEM (*n* = 19). Data are presented as mean ± SEM (*n* = 18-19). Statistical analysis was done using unpaired *t* test. **p < 0.01. **Fig. S14**. Injections of mixed cytokines resulted in decreased TH fluorescence intensity and increased Iba-1 intensity in the locus coeruleus of 3×Tg-AD mice. (a) Representative images of immunofluorescence staining for TH (green) and Iba-1 (red) in the locus coeruleus of 3×Tg-AD mice with and without mixed cytokines injection. (b) Representative images of immunofluorescence staining for TH (green) and GFAP (red) in the locus coeruleus of 3×Tg-AD mice with and without mixed cytokines injection. (c) Quantification of TH+ cells/field, TH, Iba-1, and GFAP immunofluorescence intensity in the locus coeruleus of 3×Tg-AD mice without and without mixed cytokines injection. Data are presented as mean ± SEM (*n* = 5). Statistical analysis was done using unpaired *t* test. *p < 0.05, **p < 0.01. **Fig. S15.** Total bacterial load in the whole brain homogenates of 3×TgT-AD mice at 7-day and 5-weeks postweeks post ligation measured by 16S rRNA real-time PCR.

## Data Availability

The data sets generated during and/or analyzed during the current study are available in the repository: 10.25442/hku.19779048.

## Abbreviations

AD: Alzheimer’s disease
Aβ: Beta-amyloid
ABC: Alveolar bone crest
CEJ: Cemento-enamel junction
CNS: Central nervous system
CRP: C-reactive protein
LC: Locus coeruleus
Micro-CT: Micro-computed tomography
MT: Magnetization transfer
nTg: Non-transgenic
Pg: Porphyromonas gingivalis
