# Supplementary material for: IL-1β and TNF-α play an important role in modulating the risk of periodontitis and Alzheimer’s disease
Source: J Neuroinflammation. 2023 Mar 13;20:71. doi: 10.1186/s12974-023-02747-4 (PMC10012546; doi:10.1186/s12974-023-02747-4)
Supplement: Supplementary file 1 — Additional file 1: Table S1. List of primer sequences used in this study. Fig. S1. Assessment of bone mineral density from the right mandibular jaws of C57BL/6J and 3×Tg-AD mice at 3 months, 8 months, and 15-16 months of age. (a) A representative two-dimensional sagittal image of a right mandibular jaw depicting the region of interest (highlighted in yellow) for the quantitative measurement of bone mineral density. (b) Quantitative analysis of bone mineral density. Data are presented as mean ± SEM (C57BL/6J mice: n = 5/age group, 3×Tg-AD mice: n = 9-11/age group). Statistical analysis was done using one-way ANOVA with Tukey’s post-hoc test. ***p < 0.001. Fig. S2. Periodontitis induced by the injection of heat-killed Porphyromonas gingivalis resulted in elevated CRP levels in the plasma. (a) Plasma CRP concentration in nTg mice from each group. (b) Plasma CRP concentration in 3×Tg-AD mice from each group. Data are presented as mean ± SEM (n = 5-7). Statistical analysis was done using unpaired t test. *p < 0.05. Fig. S3. Periodontitis induced by the injection of heat-killed Porphyromonas gingivalis resulted in increased phosphorylated tau proteins immunoreactivity in the brains of non-transgenic (nTg) mice. (a) Representative images of immunofluorescence staining for phosphorylated tau Ser396 (green) and DAPI (blue) in the cortex, sub-regions of the hippocampus, and thalamus of nTg mice in each group. (b) Representative images of immunofluorescence staining for phosphorylated tau Ser404 (green) and DAPI (blue) in the cortex, sub-regions of the hippocampus, and thalamus of nTg mice in each group. (c, d) Quantification of (c) phosphorylated tau Ser396 and (d) phosphorylated tau Ser404 immunofluorescence intensity in the cortex, sub-regions of the hippocampus, and thalamus of nTg mice in each group. Data are presented as mean ± SEM (n = 6). Statistical analysis was done using unpaired t test. *p < 0.05, **p < 0.01. Fig. S4. Non-transgenic (nTg) mice body weight expressed [file 12974_2023_2747_MOESM1_ESM.pdf]

Table S1

Gene	Sequences		References
MCP-1	Forward	AGCTGTAGTTTTTGTCACCAAGC	(1)
	Reverse	GACCTTAGGGCAGATGCAGT	
TNF-α	Forward	CCCCAGTCTGTATCCTTCT	(2)
	Reverse	ACTGTCCCAGCATCTTGT	
IL-1β	Forward	GATGAAGGGCTGCTTCCAAAC	(3)
	Reverse	TCCACAGCCACAATGAGTGA	
IL-6	Forward	TTCACAAGTCCGGAGAGGAG	(4)
	Reverse	TCCACGATTTCCCAGAGAAC	
IL-10	Forward	CCAAGCCTTATCGGAAATGA	(2)
	Reverse	TTCTCACCCAGGGAATTCAA	
GAPDH	Forward	ATTCAACGGCACAGTCAA	(2)
	Reverse	CTCGCTCCTGGAAGATGG	
16S rRNA	Forward	CGCTAGTAATCGTGGATCAGAATG	(5)
	Reverse	TGTGACGGGCGGTGTGTA	

Table S1 List of primer sequences used in this study.

References:

1. Uni R, Inoue T, Nakamura Y, Fukaya D, Hasegawa S, Wu CH, et al. Vagus nerve stimulation even after injury ameliorates cisplatin-induced nephropathy via reducing macrophage infiltration. Sci Rep [Internet]. 2020 Dec 1 [cited 2023 Jan 18];10(1). Available from: /pmc/articles/PMC7290038/
2. Huang C, Irwin MG, Wong GTC, Chang RCC. Evidence of the impact of systemic inflammation on neuroinflammation from a non-bacterial endotoxin animal model. J Neuroinflammation [Internet]. 2018 May 17 [cited 2023 Jan 18];15(1). Available from: /pmc/articles/PMC5960121/
3. Pettini E, Fiorino F, Cuppone AM, Iannelli F, Medaglini D, Pozzi G. Interferon-γ from Brain Leukocytes Enhances Meningitis by Type 4 Streptococcus pneumoniae. Front Microbiol [Internet]. 2015 [cited 2023 Jan 18];6(DEC):1340. Available from: /pmc/articles/PMC4664635/
4. Liu Y, Chu JMT, Yan T, Zhang Y, Chen Y, Chang RCC, et al. Short-term resistance exercise inhibits neuroinflammation and attenuates neuropathological changes in 3xTg Alzheimer’s disease mice. J Neuroinflammation [Internet]. 2020 Jan 3 [cited 2023 Jan 18];17(1):1–16. Available from: https://jneuroinflammation.biomedcentral.com/articles/10.1186/s12974-019-1653-7
5. Sato H, Yano A, Shimoyama Y, Sato T, Sugiyama Y, Kishi M. Associations of streptococci and fungi amounts in the oral cavity with nutritional and oral health status in institutionalized elders: a cross sectional study. BMC Oral Health [Internet]. 2021 Dec 1 [cited 2023 Jan 18];21(1):1–12. Available from: https://bmcoralhealth.biomedcentral.com/articles/10.1186/s12903-021-01926-0

Figure S1

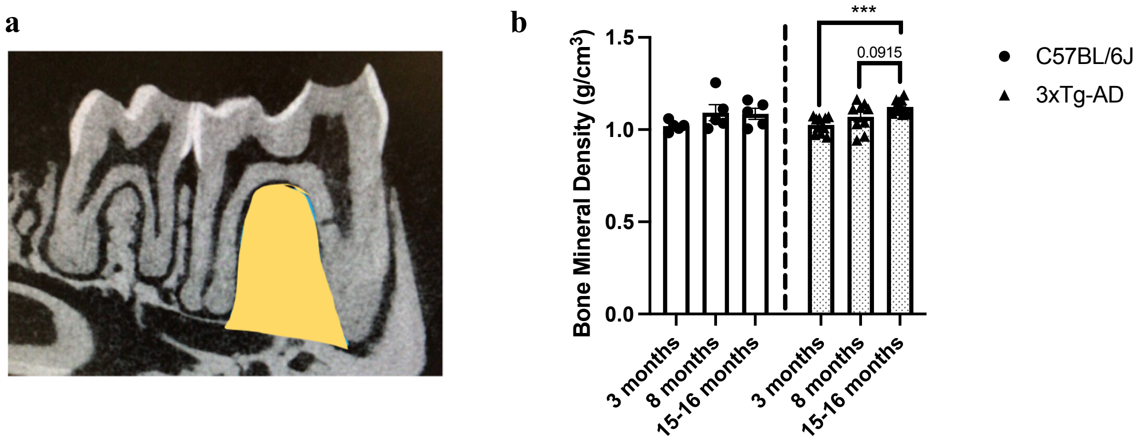

Fig. S1 Assessment of bone mineral density from the right mandibular jaws of C57BL/6J and 3×Tg-AD mice at 3 months, 8 months, and 15-16 months of age. (a) A representative two-dimensional sagittal image of a right mandibular jaw depicting the region of interest (highlighted in yellow) for the quantitative measurement of bone mineral density. **(b)** Quantitative analysis of bone mineral density. Data are presented as mean ± SEM (C57BL/6J mice: n = 5/age group, 3×Tg-AD mice: n = 9-11/age group). Statistical analysis was done using one-way ANOVA with Tukey's post-hoc test. ***p < 0.001

Figure S2

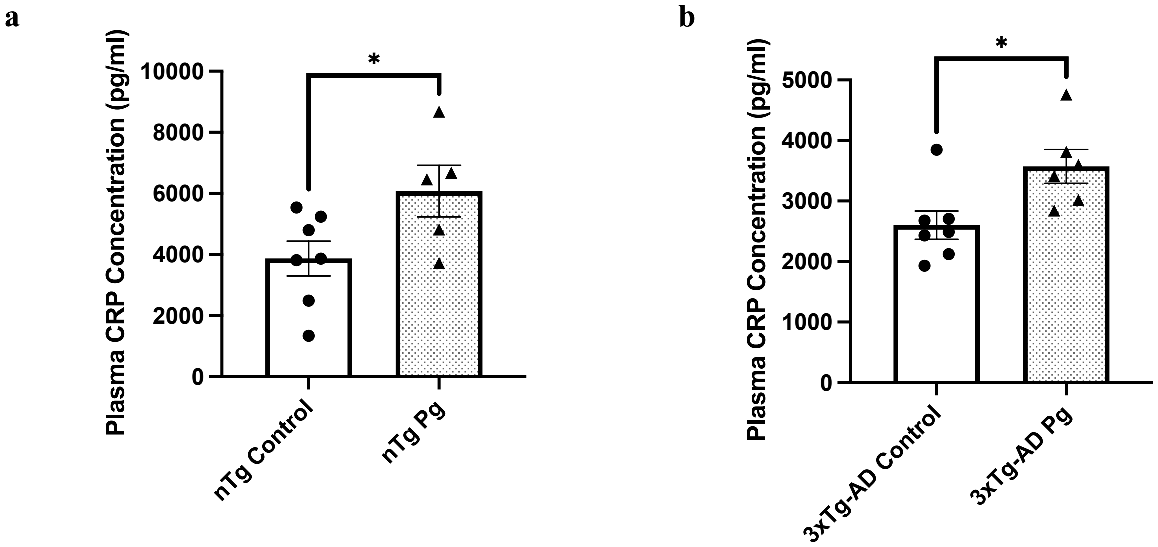

Fig. S2 Periodontitis induced by the injection of heat-killed *Porphyromonas gingivalis* resulted in elevated CRP levels in the plasma. (a) Plasma CRP concentration in nTg mice from each group. **(b)** Plasma CRP concentration in 3×Tg-AD mice from each group. Data are presented as mean ± SEM (n = 5-7). Statistical analysis was done using unpaired t test. *$p < 0.05$

Figure S3

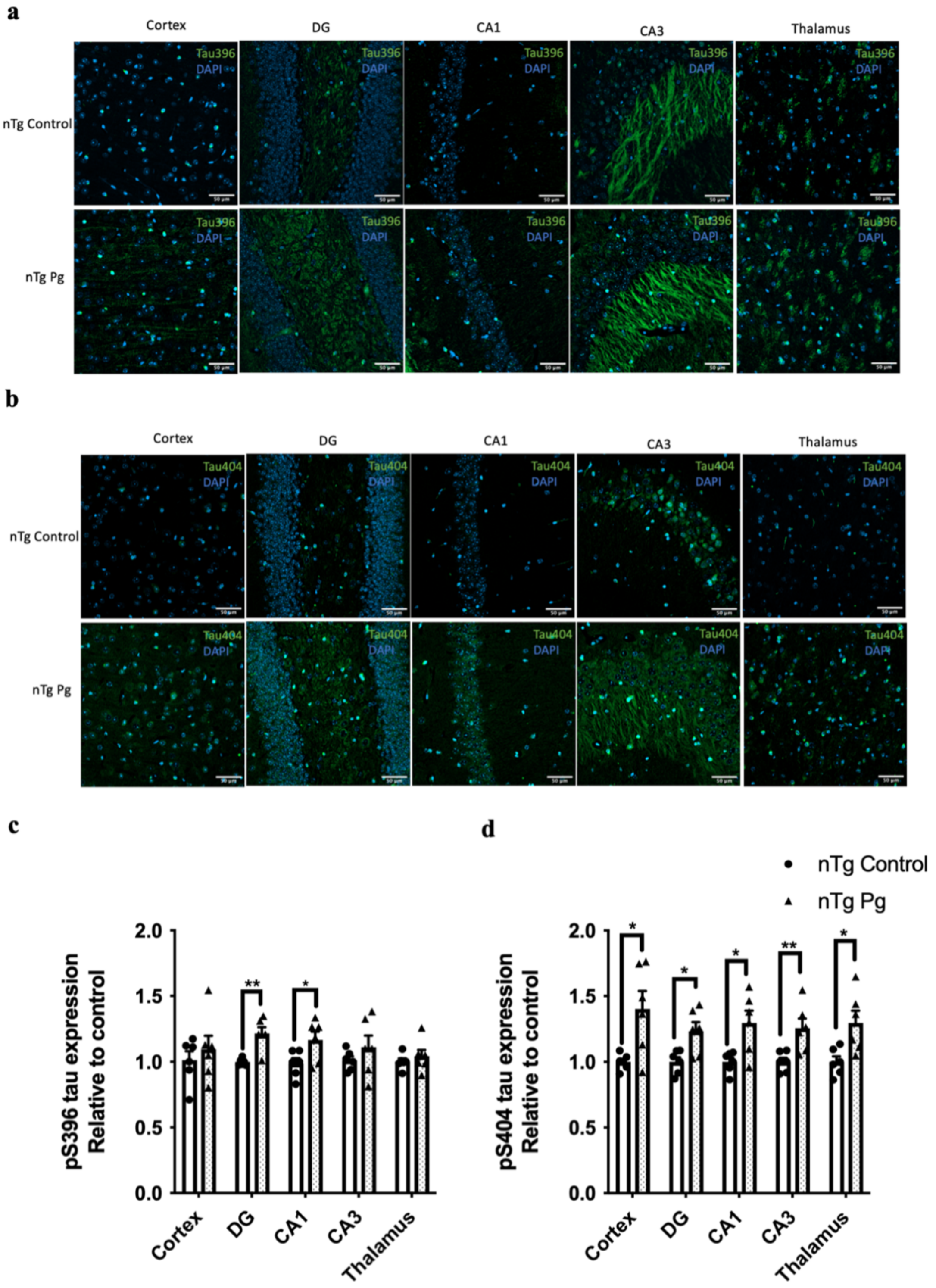
a
Cortex
DG
CA1
CA3
Thalamus
Tau396
DAPI
nTg Control
nTg Pg
b
Tau404
DAPI
c
d
nTg Control
nTg Pg
pS396 tau expression Relative to control
pS404 tau expression Relative to control
2.0
1.5
1.0
0.5
0.0
**
*

Fig. S3 Periodontitis induced by the injection of heat-killed *Porphyromonas gingivalis* resulted in increased phosphorylated tau proteins immunoreactivity in the brains of non-transgenic (nTg) mice. (a) Representative images of immunofluorescence staining for phosphorylated tau Ser396 (green) and DAPI (blue) in the cortex, sub-regions of the hippocampus, and thalamus of nTg mice in each group. **(b)** Representative images of immunofluorescence staining for phosphorylated tau Ser404 (green) and DAPI (blue) in the cortex, sub-regions of the hippocampus, and thalamus of nTg mice in each group. **(c, d)** Quantification of (c) phosphorylated tau Ser396 and (d) phosphorylated tau Ser404 immunofluorescence intensity in the cortex, sub-regions of the hippocampus, and thalamus of nTg mice in each group. Data are presented as mean ± SEM (n = 6). Statistical analysis was done using unpaired t test. $^{*}p < 0.05$, $^{**}p < 0.01$

Figure S4

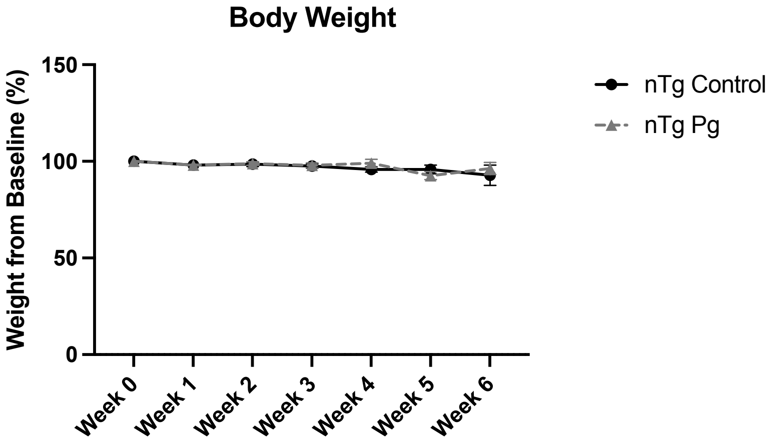

Fig. S4 Non-transgenic (nTg) mice body weight expressed as a percent change from baseline during the experimental period of heat-killed *Porphyromonas gingivalis* injections. Data are presented as mean ± SEM (n = 7-8). Statistical analysis was done using two-way ANOVA with Bonferroni's post-hoc test.

Figure S5

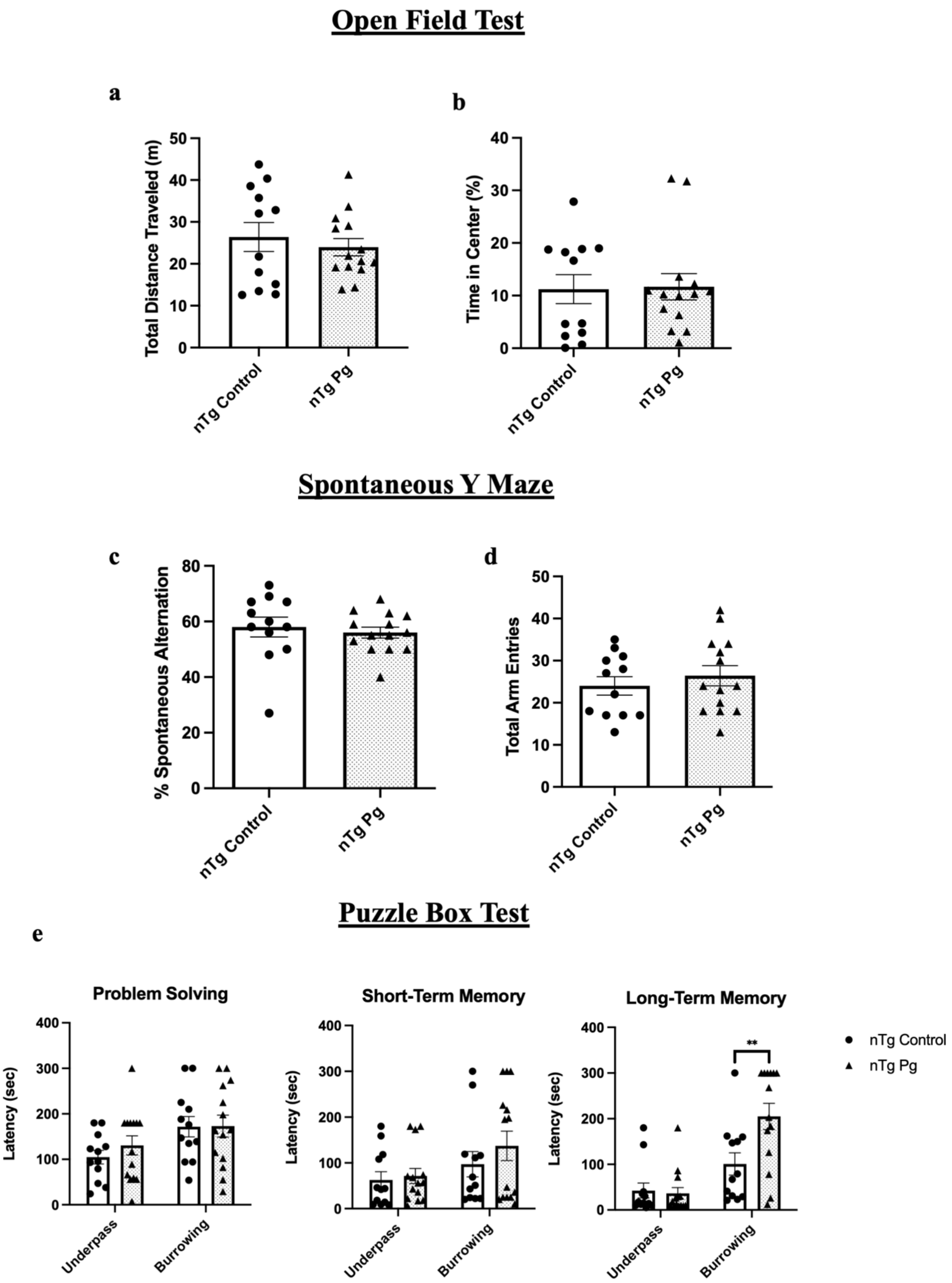
Open Field Test
a
Total Distance Traveled (m)
50
40
30
20
10
0
nTg Control
nTg Pg
b
Time in Center (%)
40
30
20
10
0
nTg Control
nTg Pg
Spontaneous Y Maze
c
% Spontaneous Alternation
80
60
40
20
0
nTg Control
nTg Pg
d
Total Arm Entries
50
40
30
20
10
0
nTg Control
nTg Pg
Puzzle Box Test
e
Problem Solving
Short-Term Memory
Long-Term Memory
Latency (sec)
400
300
200
100
0
Underpass
Burrowing
**
nTg Control
nTg Pg

Fig. S5 Periodontitis induced by the injection of heat-killed *Pg* resulted in impairment of long-term memory in non-transgenic (nTg) mice. (a, b) Open field test for the assessment of general exploration and anxiety behavior. (a) Total distance traveled and (b) the percentage of time spent in center were examined. **(c, d)** Spontaneous Y maze test for the assessment of short-term spatial working memory. (c) Percentage spontaneous alternation and (d) the number of total arm entries were examined. **(e)** Puzzle box test for the assessment of problem-solving abilities and executive functions. Data are presented as mean ± SEM (n = 12-14). Statistical analysis was done using unpaired t test. $^{**}p < 0.01$

Figure S6

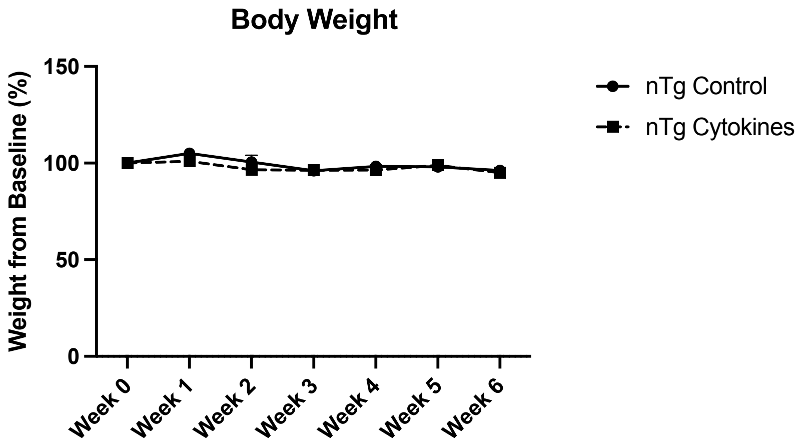

Fig. S6 Non-transgenic (nTg) mice body weight expressed as a percent change from baseline during the experimental period of mixed cytokines injections. Data are presented as mean ± SEM (n = 7). Statistical analysis was done using two-way ANOVA with Bonferroni's post-hoc test.

Figure S7

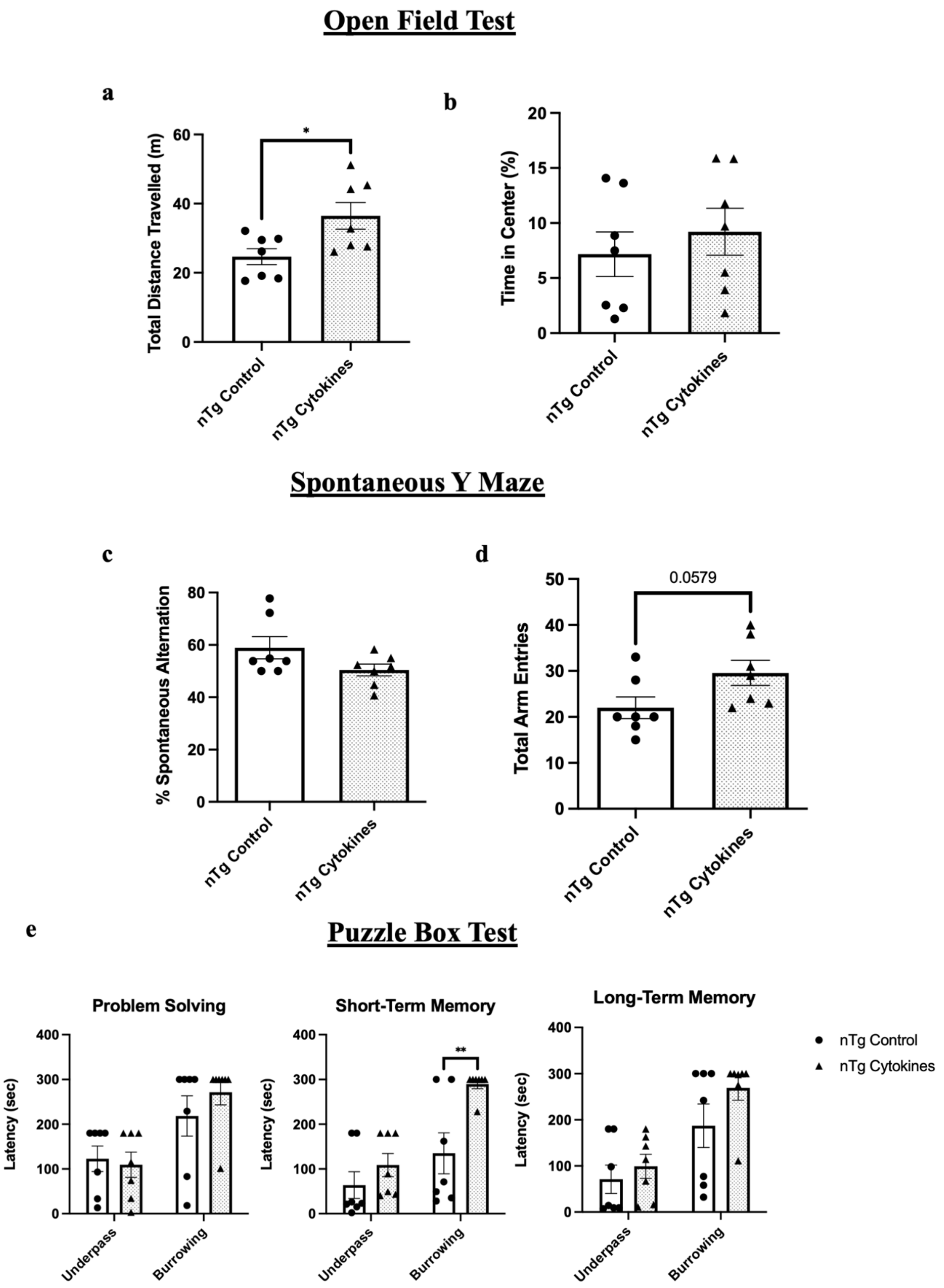
Open Field Test
a
Total Distance Travelled (m)
60
40
20
0
*
nTg Control
nTg Cytokines
b
Time in Center (%)
20
15
10
5
0
nTg Control
nTg Cytokines
Spontaneous Y Maze
c
% Spontaneous Alternation
80
60
40
20
0
nTg Control
nTg Cytokines
d
Total Arm Entries
50
40
30
20
10
0
0.0579
nTg Control
nTg Cytokines
e
Puzzle Box Test
Problem Solving
Short-Term Memory
Long-Term Memory
Latency (sec)
400
300
200
100
0
**
Underpass
Burrowing
nTg Control
nTg Cytokines

Fig. S7 Injections of mixed cytokines resulted in hyperactivity and impairment of short-term memory in non-transgenic (nTg) mice. (a, b) Open field test for the assessment of general exploration and anxiety behavior. (a) Total distance traveled and (b) the percentage of time spent in center were examined. **(c, d)** Spontaneous Y maze test for the assessment of short-term spatial working memory. (c) Percentage spontaneous alternation and (d) the number of total arm entries were examined. **(e)** Puzzle box test for the assessment of problem-solving abilities and executive functions. Data are presented as mean ± SEM (n = 7). Statistical analysis was done using unpaired t test. *$^{*}p < 0.05$, $^{**}p < 0.01$*

Figure S8

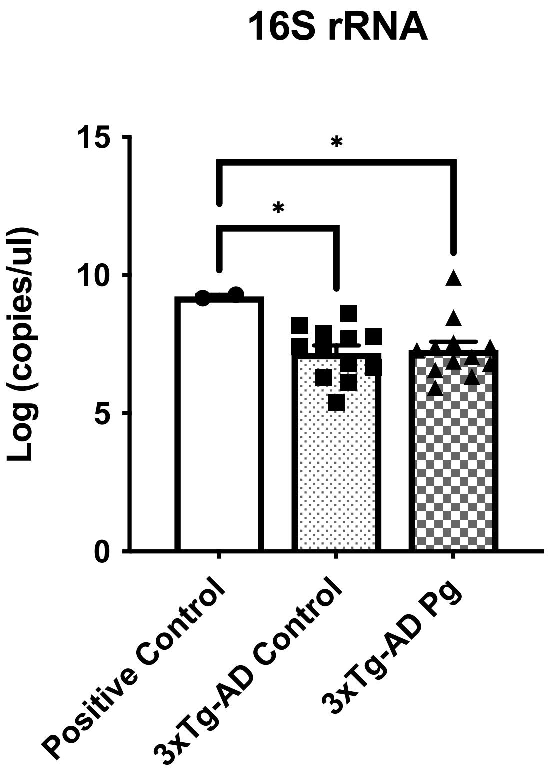

Fig. S8 Total bacterial load in the gums of 3×Tg-AD mice measured by 16S rRNA real-time PCR at the end of the experimental period. Tissue DNA spiked with 100 ng of Pg DNA was included as positive controls. Data are presented as mean ± SEM (n = 12). Statistical analysis was done using one-way ANOVA with Tukey's post-hoc test. *$p < 0.05$

Figure S9

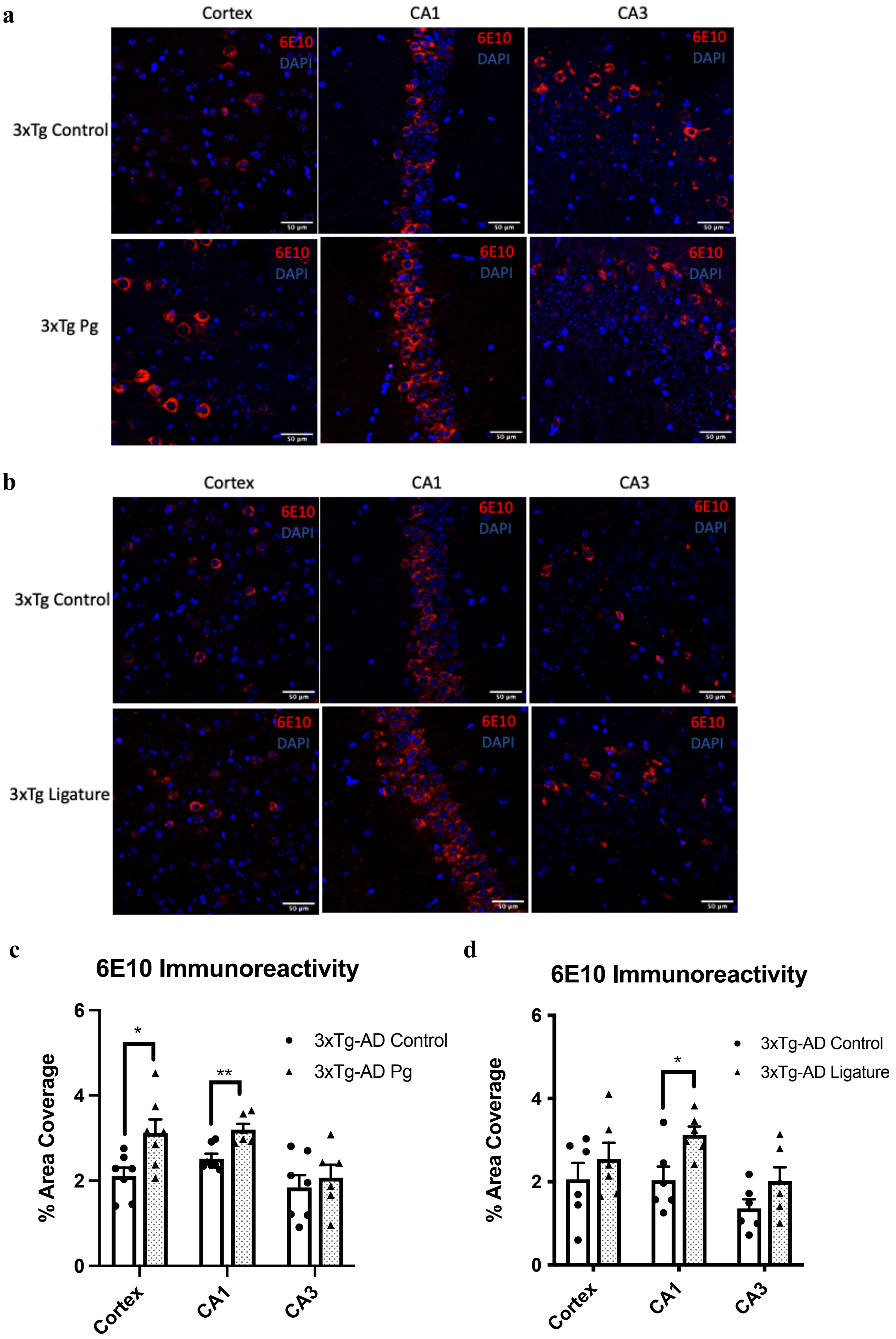
a
Cortex
CA1
CA3
6E10
DAPI
3xTg Control
3xTg Pg
b
Cortex
CA1
CA3
6E10
DAPI
3xTg Control
3xTg Ligature
c
6E10 Immunoreactivity
% Area Coverage
0
2
4
6
*
**
3xTg-AD Control
3xTg-AD Pg
Cortex
CA1
CA3
d
6E10 Immunoreactivity
% Area Coverage
0
2
4
6
*
3xTg-AD Control
3xTg-AD Ligature
Cortex
CA1
CA3

Fig. S9 Periodontitis induced by injection of heat-killed *Pg* as well as ligature placement resulted in increased 6E10 immunoreactivity in the brains of 3×Tg-AD mice. (a-b) Representative images of immunofluorescence staining for 6E10 (red) and DAPI (blue) in the cortex and sub-regions of hippocampus of 3×Tg-AD mice with and without (a) *Pg* injections and (b) ligature placement. **(c-d)** Quantification of 6E10 immunofluorescence intensity in the cortex and sub-regions of the hippocampus of 3×Tg-AD mice with and without (c) *Pg* injections and (d) ligature placement. Data are presented as mean ± SEM (n = 6-7). Statistical analysis was done using unpaired t test. $^{*}p < 0.05$, $^{**}p < 0.01$

Figure S10

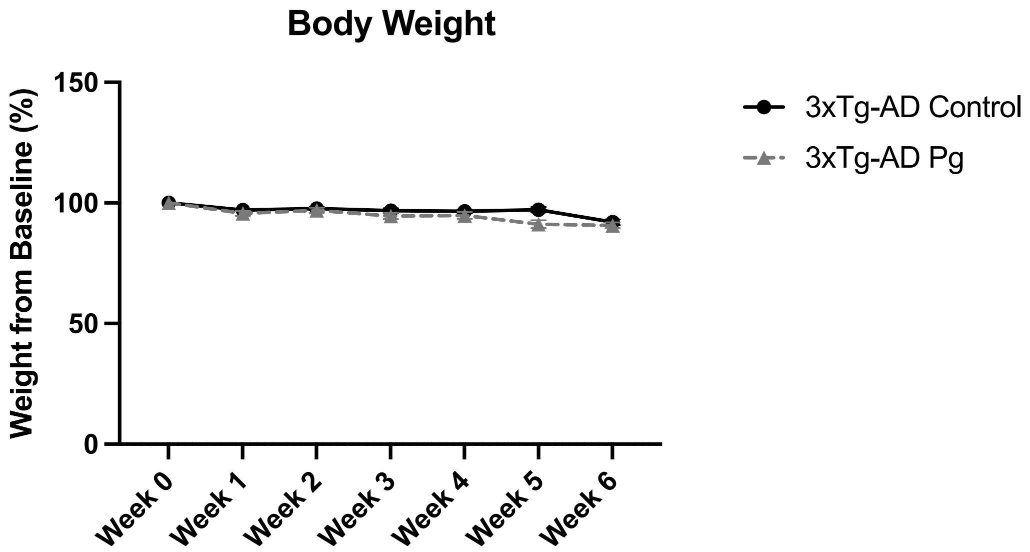

Fig. S10 3×Tg-AD mice body weight expressed as a percent change from baseline during the experimental period of heat-killed *Pg* injections. Data are presented as mean ± SEM (n = 7-8). Statistical analysis was done using two-way ANOVA with Bonferroni's *post-hoc* test.

Figure S11

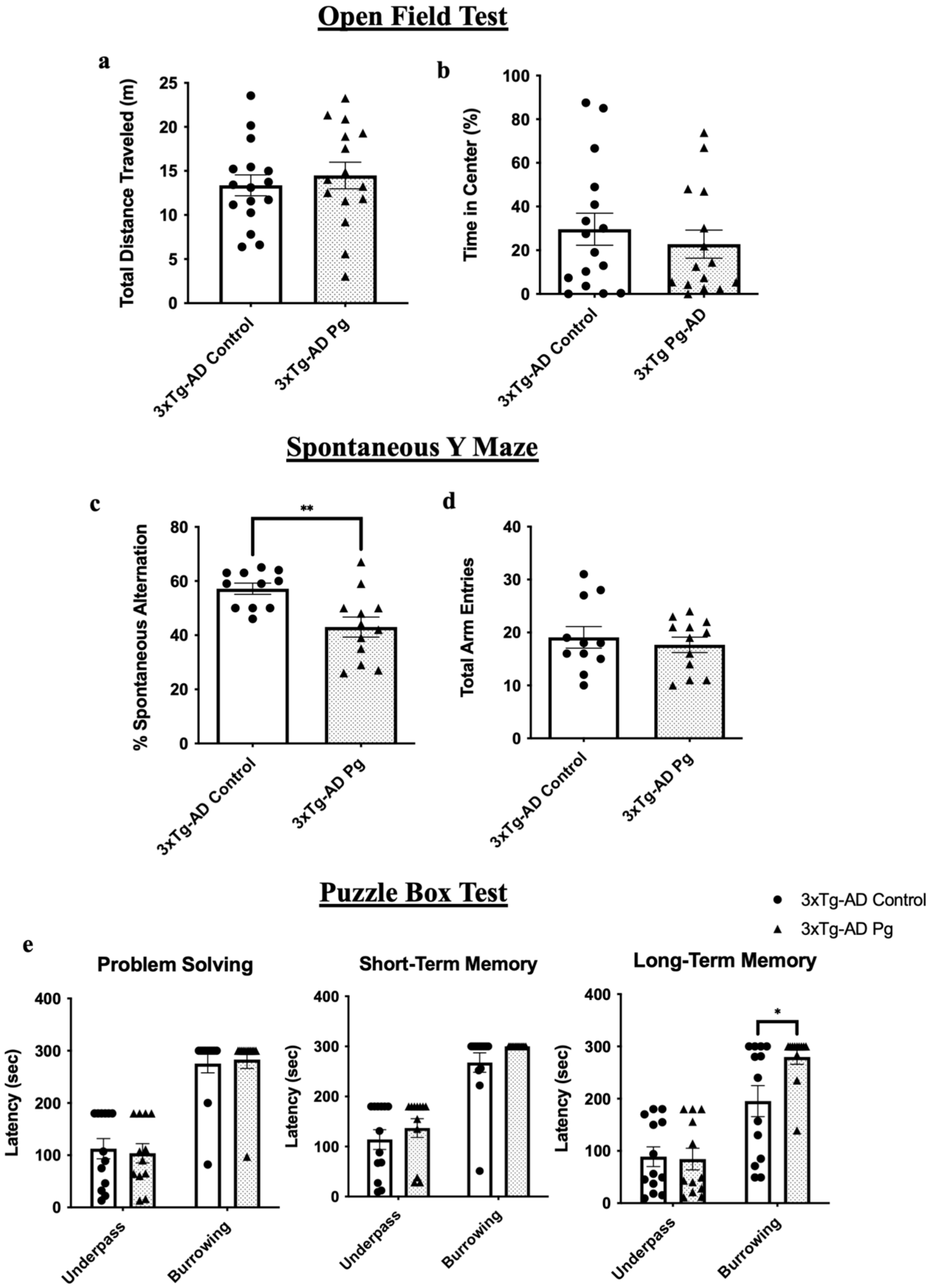
Open Field Test
a
Total Distance Traveled (m)
3xTg-AD Control
3xTg-AD Pg
b
Time in Center (%)
3xTg-AD Control
3xTg Pg-AD
Spontaneous Y Maze
c
**
% Spontaneous Alternation
3xTg-AD Control
3xTg-AD Pg
d
Total Arm Entries
3xTg-AD Control
3xTg-AD Pg
Puzzle Box Test
3xTg-AD Control
3xTg-AD Pg
e
Problem Solving
Short-Term Memory
Long-Term Memory
*
Latency (sec)
Underpass
Burrowing

Fig. S11 Periodontitis induced by the injection of heat-killed *Porphyromonas gingivalis* resulted in impairment of both short- and long-term memory in 3×Tg-AD mice. (a, b) Open field test for the assessment of general exploration and anxiety behavior. (a) Total distance traveled and (b) the percentage of time spent in center were examined. Data are presented as mean ± SEM (n = 15-16). **(c, d)** Spontaneous Y maze test for the assessment of short-term spatial working memory. (c) Percentage spontaneous alternation and (d) the number of total arm entries were examined. Data are presented as mean ± SEM (n = 11-12). (e) Puzzle box test for the assessment of problem-solving abilities and executive functions. Data are presented as mean ± SEM (n = 12-13). Statistical analysis was done using unpaired t test. $^{*}p < 0.05$, $^{**}p < 0.01$

Figure S12

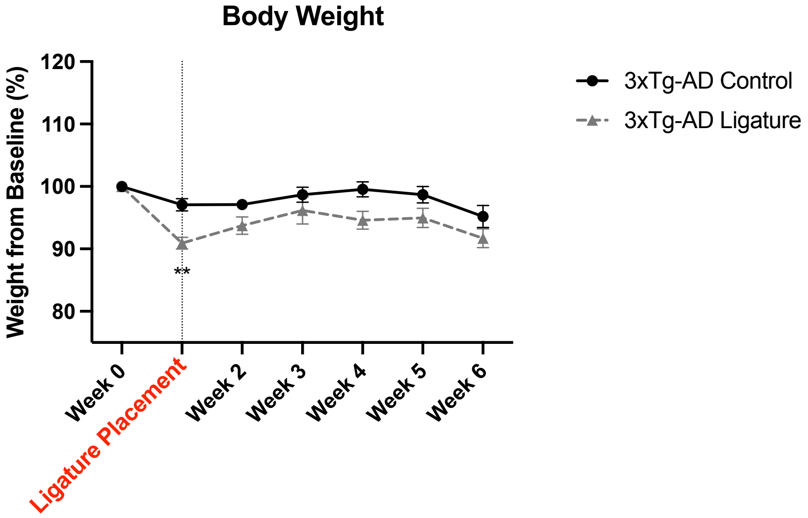

Fig. S12 3×Tg-AD mice body weight expressed as a percent change from baseline during the experimental period of ligature placement. Data are presented as mean ± SEM (n = 12). Statistical analysis was done using two-way ANOVA with Bonferroni's *post-hoc* test. $^{**}p < 0.01$

Figure S13

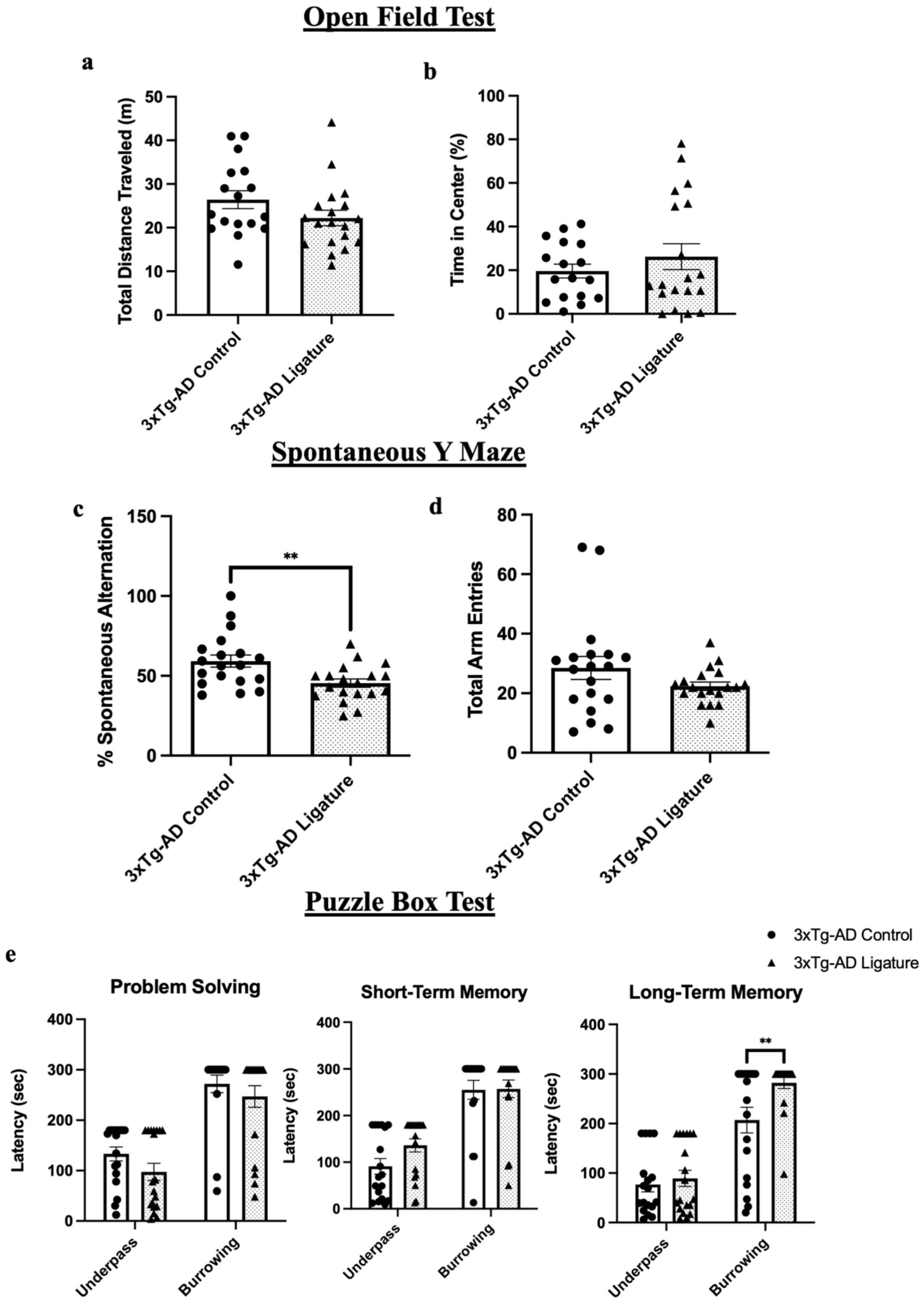
Open Field Test
a
Total Distance Traveled (m)
3xTg-AD Control
3xTg-AD Ligature
b
Time in Center (%)
3xTg-AD Control
3xTg-AD Ligature
Spontaneous Y Maze
c
% Spontaneous Alternation
**
3xTg-AD Control
3xTg-AD Ligature
d
Total Arm Entries
3xTg-AD Control
3xTg-AD Ligature
Puzzle Box Test
e
3xTg-AD Control
3xTg-AD Ligature
Problem Solving
Short-Term Memory
Long-Term Memory
Latency (sec)
**
Underpass
Burrowing

Fig. S13 Ligature-induced periodontitis resulted in impairment of both short- and long-term memory in 3×Tg-AD mice. (a, b) Open field test for the assessment of general exploration and anxiety behavior. (a) Total distance traveled and (b) the percentage of time spent in center were examined. Data are presented as mean ± SEM (n = 17-19). **(c, d)** Spontaneous Y maze test for the assessment of short-term spatial working memory. (c) Percentage spontaneous alternation and (d) the number of total arm entries were examined. Data are presented as mean ± SEM (n = 19). Data are presented as mean ± SEM (n = 18-19). Statistical analysis was done using unpaired t test. ***$p < 0.01$*

Figure S14

a

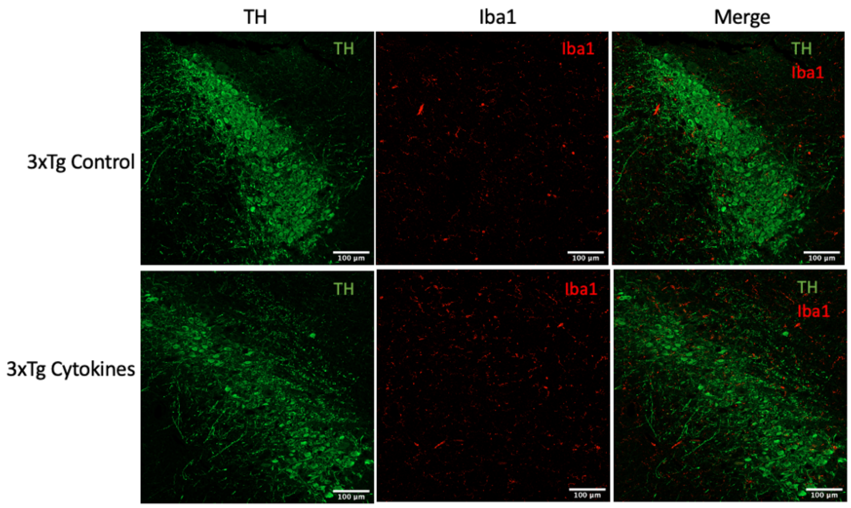

b

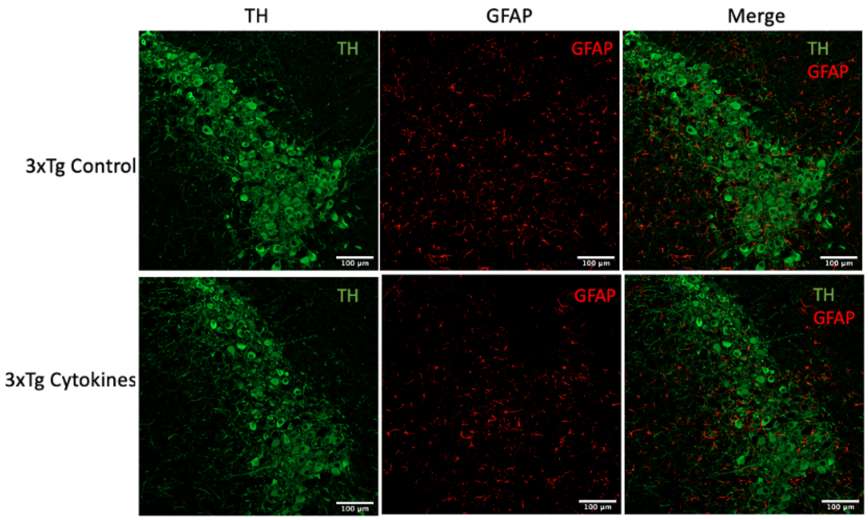

c

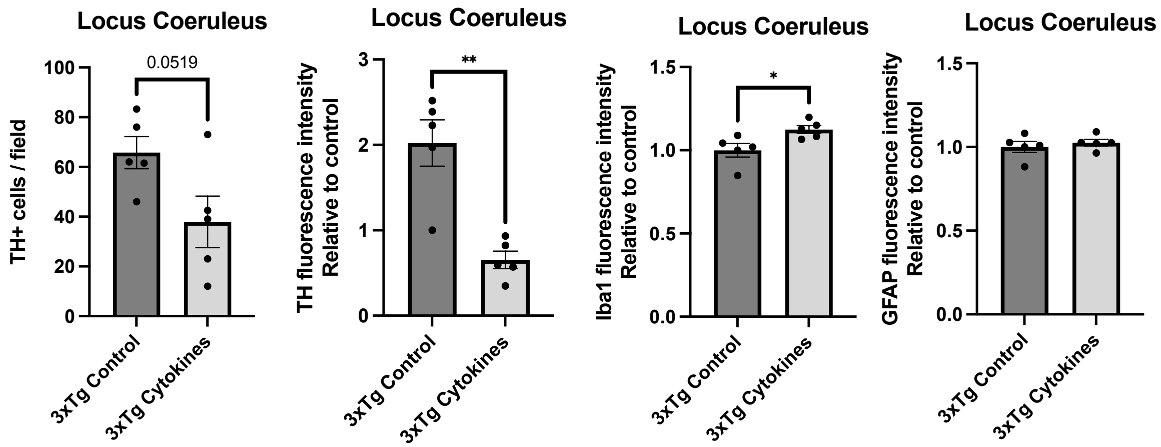

Fig. S14 Injections of mixed cytokines resulted in decreased TH fluorescence intensity and increased Iba-1 intensity in the locus coeruleus of 3×Tg-AD mice. (a) Representative images of immunofluorescence staining for TH (green) and Iba-1 (red) in the locus coeruleus of 3×Tg-AD mice with and without mixed cytokines injection. **(b)** Representative images of immunofluorescence staining for TH (green) and GFAP (red) in the locus coeruleus of 3×Tg-AD mice with and without mixed cytokines injection. **(c)** Quantification of TH+ cells/field, TH, Iba-1, and GFAP immunofluorescence intensity in the locus coeruleus of 3×Tg-AD mice without and without mixed cytokines injection. Data are presented as mean ± SEM (n = 5). Statistical analysis was done using unpaired t test. *$p < 0.05$, **$p < 0.01$

Figure S15

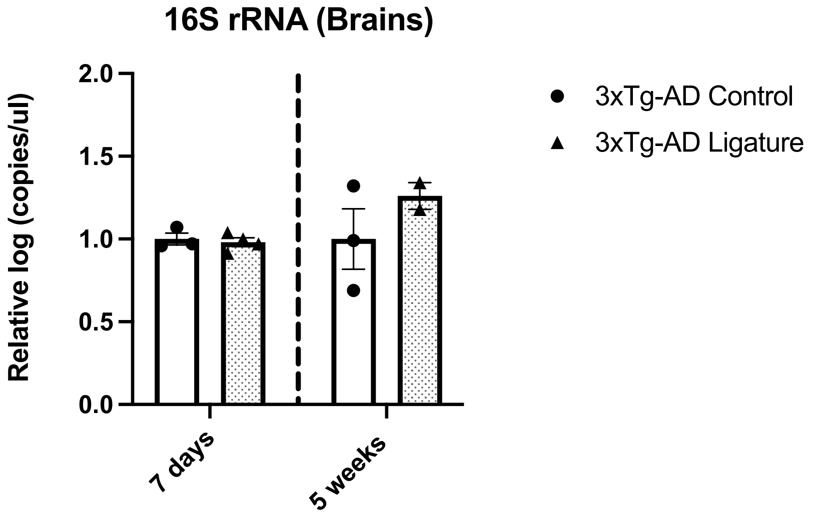

Fig. S15 Total bacterial load in the whole brain homogenates of 3×Tg-AD mice at 7-day and 5-weeks post-ligation measured by 16S rRNA real-time PCR. Data are presented as mean ± SEM (n = 2-4). Statistical analysis was done using unpaired t test.
